# Expressed Centromere Specific Histone 3 (*CENH3*) Variants in Cultivated Triploid and Wild Diploid Bananas (*Musa* spp.)

**DOI:** 10.3389/fpls.2017.01034

**Published:** 2017-06-29

**Authors:** Kariuki S. Muiruri, Anne Britt, Nelson O. Amugune, Edward K. Nguu, Simon Chan, Leena Tripathi

**Affiliations:** ^1^International Institute of Tropical AgricultureNairobi, Kenya; ^2^School of Biological Sciences, University of NairobiNairobi, Kenya; ^3^Department of Plant Biology, University of California, Davis, DavisCA, United States; ^4^Department of Biochemistry, University of NairobiNairobi, Kenya

**Keywords:** *CENH3*, splice variants, genotype, centromere, histones, banana

## Abstract

Centromeres are specified by a centromere specific histone 3 (CENH3) protein, which exists in a complex environment, interacting with conserved proteins and rapidly evolving satellite DNA sequences. The interactions may become more challenging if multiple CENH3 versions are introduced into the zygote as this can affect post-zygotic mitosis and ultimately sexual reproduction. Here, we characterize *CENH3* variant transcripts expressed in cultivated triploid and wild diploid progenitor bananas. We describe both splice- and allelic-[Single Nucleotide Polymorphisms (SNP)] variants and their effects on the predicted secondary structures of protein. Expressed *CENH3* transcripts from six banana genotypes were characterized and clustered into three groups (*MusaCENH*-1A, *MusaCENH*-1B, and *MusaCENH*-2) based on similarity. The *CENH3* groups differed with SNPs as well as presence of indels resulting from retained and/or skipped exons. The *CENH3* transcripts from different banana genotypes were spliced in either 7/6, 5/4 or 6/5 exons/introns. The 7/6 and the 5/4 exon/intron structures were found in both diploids and triploids, however, 7/6 was most predominant. The 6/5 exon/introns structure was a result of failure of the 7/6 to splice correctly. The various transcripts obtained were predicted to encode highly variable N-terminal tails and a relatively conserved C-terminal histone fold domain (HFD). The SNPs were predicted in some cases to affect the secondary structure of protein by lengthening or shorting the affected domains. Sequencing of banana *CENH3* transcripts predicts SNP variations that affect amino acid sequences and alternatively spliced transcripts. Most of these changes affect the N-terminal tail of CENH3.

## Introduction

Centromeres are assembly sites for the kinetochore, a protein complex that connects chromosomes to spindle fibers during meiosis and mitosis. The structure, size, and distribution of centromeres differ with species in spite of their common function (Talbert et al., 2004). Centromeres in both plants and animals often contain arrays of rapidly evolving tandemly repeated DNA sequences (Gent et al., 2011; Verdaasdonk and Bloom, 2011). The high rate of evolution in these repeats is remarkable given the fact that the function of centromeres is highly conserved. The role of the repeats is a subject of debate with the most common proposition being that they maintain the large heterochromatic domains associated with centromeres (Malik and Henikoff, 2009; Black and Cleveland, 2011). It is reported that CENH3 [aka Centromere Protein A (CENP-A) in humans and CID in drosophila] epigenetically determines and maintains centromeres (Malik and Henikoff, 2001; Dawe and Henikoff, 2006; Ekwall, 2007; Allshire and Karpen, 2008; Fachinetti et al., 2013). CENH3 contains a highly variable N-terminal tail and a relatively conserved histone fold domain (HFD) (Ravi et al., 2010; Lermontova et al., 2014). The majority of diploid plant species have been shown to encode a single *CENH3* gene (Zhong et al., 2002). However, more than one copy (alpha and beta) of the gene per genome are present in some species like wheat, barley, *Arabidopsis halleri* and *A. lyrata* (Kawabe et al., 2006; Sanei et al., 2011; Yuan et al., 2015).

The majority of cultivated bananas exist as allo- or autopolyploids and a variety of *CENH3* isoforms are presumed to coexist in the nucleus. Polyploidization brings together multiple gene copies within the same background and can result in additive or non-additive gene expression leading to biased or unbiased homeolog expression (Pignatta and Comai, 2009; Hui et al., 2010; Rapp et al., 2010; Yoo et al., 2013). Unlike many diploid species where a single copy of *CENH3* gene is encoded, multiple copies have been observed in newly synthesized allopolyploids of rice, wheat, brassica, and pea (Hirsch et al., 2009; Hui et al., 2010; Wang et al., 2011; Neumann et al., 2012; Yuan et al., 2015). *CENH3* variants have also been characterized in wild and cultivated carrots (Dunemann et al., 2014) and in stable polyploids of different angiosperms (Masonbrink et al., 2014). Multiple *CENH3* copies observed in polyploids might result from coming together of single-*CENH3*-expressing genomes or multiple-*CENH3* expressing progenitor genomes. Crosses of diploid parents encoding multiple *CENH3* transcripts have resulted in stable hybrids. For example, in stable hybrids from *Hordeum vulgare* × *H. bulbosum* crosses, both alpha and beta *CENH3* variants from *H. vulgare* were incorporated into the centromeric nucleosomes of the hybrid. In contrast, a hybrid of *H. bulbosum* × *Triticum aestivum* incorporated the *H. bulbosum CENH3* variant HbαCENH3 only (Sanei et al., 2011).

Unlike stable hybrids, embryos derived from unstable crosses have been observed to undergo uniparental genome elimination, resulting in haploids carrying genetic material from only one parent (Ravi and Chan, 2010; Sanei et al., 2011; Seymour et al., 2012; Maheshwari et al., 2015). The genome of *H. bulbosum* in embryos from *H. vulgare* × *H. bulbosum* crosses for example was completely lost within 5–9 days post-fertilization. Despite elimination of the *H. bulbosum* genome later in post-zygotic mitosis, *H. vulgare* × *H. bulbosum* unstable crosses have been observed to transcribe *CENH3* transcript variants from both parents (Sanei et al., 2011). In *A. thaliana*, uniparental genome elimination was also observed in offspring from crosses between mutant ‘haploid inducer’ (parent with modified *CENH3*) and wild-type (carrying wild-type *CENH3* version) (Ravi and Chan, 2010). The modification of *CENH*3 in this case was generated by replacing the N-terminal tail with that of the variant H3.3 and tagging it with GFP. Apart from obtaining haploids in these crosses, novel genetic rearrangements were observed (Maheshwari et al., 2015). Currently, there are efforts undergoing to transfer this technology to many crops including banana (Comai, 2014). Crosses of *A. thaliana* null-mutants carrying gene constructs expressing *CENH3* from distant species to plants wild-type for *CENH3* have also resulted in haploids (Maheshwari et al., 2015). Furthermore, uniparental genome elimination has been observed in crosses of wild-type *A. thaliana* plants to null mutants complemented with *CENH3* carrying missense point mutations in conserved regions of the HFD (Kuppu et al., 2015).

Banana breeding involves crossing of tetraploids to diploids to give triploids and this may add into the complexity of the space CENH3 exists. Therefore, it would be interesting and useful to understand *CENH3* dynamics in cultivated polyploids and their diploid progenitors. Furthermore, a clear understanding of *CENH3* behavior in cultivated crops like banana is essential if breeding tools such as *CENH3*-based haploid technology are to be effectively applied (Britt and Kuppu, 2016). Therefore, in this study the expression of *CENH*3 was characterized in cultivated triploid and wild-type diploid progenitor bananas. The existence and evolutionary relationships of *CENH3* SNPs and/or splice variants as well as their predicted secondary folding of protein were analyzed.

## Materials and Methods

### Plant Materials

Six banana genotypes including wild diploids ‘Calcutta 4’ (AA) and ‘Zebrina GF’ (AA) both from the species *Musa acuminata*, the species *M. balbisiana* (BB) and cultivated triploids ‘Sukali Ndiizi’ (AAB), ‘Pisang Awak’ (ABB) and ‘Gros Michel’ (AAA) were used in this study. All plant materials used were obtained from *in vitro* collection at IITA Kenya.

### Identification of Genomic Sequence of Banana *CENH3*

To identify putative genomic sequence of banana *CENH3*, a nucleotide BLAST (BLASTN) analysis was performed using genomic sequence of *A. thaliana CENH3* (At1g01030) against the whole-genome shotgun contigs (wgs) of *M. acuminata* (tax id: 4641) for “somewhat similar sequences”. In order to identify the exact genomic region of *CENH3*, consensus sequences from conserved regions at the beginning and end of selected monocot *CENH3* CDSs were mapped to the BLASTN hit results. The conserved consensus, which we considered as representative *CENH3* ‘landmark’ regions for monocots, were obtained by aligning sequences of *CENH3* from the monocots *Zea mays* (NM_001112050), *H. vulgare* (JF419328), *T. aestivum* (JF969285.1) and *Oryza sativa* (AY438639.1). To identify the genomic regions of the *CENH3* from *M. acuminata*, BLASTN hits, we mapped the *CENH3* ‘landmarks’ and regions with >75% nucleotide identities were selected. The primers CENH3_END_F (GGCGAGAACGAAGCATC) and CENH3_END_R (TCACCAATGTCTTCTTCCTCC) were designed to amplify the CDS (from the beginning to the end of the coding region) derived from *in silico* analysis of the putative banana genomic sequence (Accession: CAIC01023700).

### RNA Extraction and RT-PCR

Total RNA was extracted from 100 mg of young incompletely open leaves. Extraction was performed using RNeasy^®^ plant mini kit (Hilden, Germany) as per the manufacturer’s protocol except for the elution volume which was reduced to 40 μl. Genomic DNA contamination was removed from the extracted RNA through DNase I (Thermo Scientific, Waltham, MA, United States) treatment by incubating at 37°C for 30 min and then terminating the reaction by adding 1 mM EDTA and heating at 70°C for 5 min. RNA quality and quantity were checked using a NanoDrop^TM^ 2000 (Thermo Scientific, Waltham, MA, United States) spectrophotometer.

First strand cDNA was synthesized from 1 μg of DNA-free total RNA with random hexamer primers using maxima first strand reverse transcriptase kit (Thermo Scientific, Waltham, MA, United States). Two independent cDNA synthesis reactions were performed for each of the genotype.

The *CENH3* transcripts were amplified from cDNA in a total of six PCR reactions (three reactions for each of the two cDNA synthesis) per genotype. Each PCR reaction was performed in a 20 μl volume, which contained 50 ng of cDNA template, 1x Q5 reaction buffer containing 2.5 mM MgCl_2_, 500 μM of each dNTP, 10 μM each of *CENH3* primers (CENH3_END_F and CENH3_END_R) and 1unit of Q5 high fidelity DNA polymerase (New England Biolabs, MA). The reactions were performed in an ABI 9700 PCR machine with the conditions set at initial denaturation of 98°C for 4 min, 35 cycles of 98°C for 15 s, 66°C for 30 s and 72°C for 45 s and a final extension at 72°C for 10 min. An aliquot of PCR product (2 μl) was run on a 1.5% agarose gel stained with GelRed (Biotium, CA) to confirm amplification. For PCR reactions in each genotype that had observable band(s) on agarose gel, the remainder (18 μl) PCR product was purified using Bioneer PCR purification kit (Daeongeon, South Korea) and eluted in 15 μl water.

### Cloning and Sequencing of *CENH3* Genes

Purified PCR products were cloned into pJET 1.2 cloning vector (Thermo Scientific, MA) and transformed into competent *Escherichia coli* (DH5α) cells using heat shock method. The transformed *E. coli* colonies were selected on Luria Bertani (LB) agar (10 g/l Tryptone, 5 g/l Yeast extract, 10 g/l NaCl, 15 g/l Agar, pH 7.5) containing 50 mg/L ampicillin. One to 10 transformed colonies from each PCR reaction were screened for presence of the insert by colony-PCR. A maximum of 60 colonies were screened for each genotype. The primer pairs pJET 1.2_F: CGACTCACTATAGGGAGAGCGGC and pJET 1.2_R: AAGAACATCGATTTTCCATGGCAG were used for colony PCR. Colonies with amplicon sizes >200 bp were cultured in LB broth medium overnight at 37°C and plasmid DNA extracted using Qiagen plasmid miniprep kit. Each clone with product >200 bp was sequenced bi-directionally in three replicates using the primers pJET 1.2_F and Pjet 1.2_R. Sequencing was performed on ABI 3130 analyzer (Applied Biosystems, Foster City, CA, United States) using BigDye Terminator Kit version 3.1.

### Sequence Analysis and Multiple Alignments

Sequences were analyzed in Geneious version 7.1 (Biomatter, NZ) (Kearse et al., 2012) by manually checking the quality of the chromatograms. Sequences with quality above 50% (based on Phred values) across the entire sequence length were used for analysis. Sequences were further screened and ‘dirty’ sections at the ends were manually trimmed to retain only high quality regions. Sequences within any of the six genotypes that were independently derived (those obtained from amplification of independently synthesized cDNA transcripts) and had 100% similarity were considered to represent the same transcript.

Multiple alignments of amino acids were conducted among translated banana CENH3 sequences and monocots (*Z. mays, T. aestivum, O. sativa*, and *H. vulgare*) and dicots (*A. thaliana and Brassica rapa*) in MUSCLE as implemented in Geneious version 7.1 using default parameters. Phylogenetic trees comparing transcript sequences were drawn in the software “Molecular and Evolutionary Genetic Analysis” (MEGA) version 6.0 (Tamura et al., 2013) based on only the conserved tail sections and entire HFD region.

### Identification of Exon/Intron Structures

Since the banana *CENH3* from the genotypes used in this study had not been sequenced previously, splicing patterns for the transcript sequences were predicted by aligning them to the then available banana genomic sequence (accession number: CAIC01023700 positions 70772 to 76310) from *M. acuminata* genotype ‘DH Pahang’ using the program Splign (Kapustin et al., 2008).

### Protein Structure Modeling

Secondary structures of proteins were predicted using the original Garnier Osguthorpe Robson algorithm (GOR I) provided by the European Molecular Biology Open Software Suite (EMBOSS) 6.5.7 (Rice et al., 2000) and implemented in Geneious version 7.1.9 as garnier tool (Kearse et al., 2012). Predicted protein structures from transcripts of different length, SNP and splice were visually compared to determine any variation in their secondary folding.

## Results

### Identification of Genomic Sequence of Banana *CENH3*

To identify *CENH3* genomic sequence from completely sequenced banana genome [doubled haploid (DH) genotype ‘DH Pahang’ (‘Malaccensis’ group)] (Hont et al., 2012), a BLASTN was performed for ‘somewhat’ similar targets using *A. thaliana CENH3* to query *M. acuminata* whole-genome contigs. This search resulted in a total of 46 hits (**Additional File S1**). To identify the exact banana *CENH3* genomic region(s), conserved consensus sequences at the beginning (ATGGCSMGMACSAAGCAYCCGGCSGTGMGSAARAGC) and end (GCAAGGCGWATMGGAGGRAGRAGRCATTGGTGATGA) of *CENH3* CDSs from four monocotyledonous plants (rice, maize, barley, and millet), referred as monocot *CENH3 ‘*landmarks’, were searched within the 46 BLASTN hits. A search for these consensus sequences within the 46 BLASTN hit revealed an 82 Kb contiguous sequence (GenBank accession number: CAIC01023700) as containing the putative banana *CENH3* genomic region. The exact location of the sequence within the 82 Kb contig CAIC01023700 was from positions 70772 to 76310 resulting in a 5538 bp long sequence.

### Banana *CENH3* Sequences and Expressed Variants

In an effort to identify banana *CENH3* transcripts in each of the six banana genotypes, PCR products from amplification of cDNA template obtained from two independent synthesis reactions were cloned and sequenced. One to seven unique transcripts were obtained per genotype by sequencing of the multiple clones. The multiple clones sequenced were derived from three independent PCR amplifications of the two cDNA templates for a maximum of six reactions per genotype (**Table 1**). The genotype ‘Calcutta 4’ and ‘*M. balbisiana*’ had only one unique sequence each, where as ‘Gros Michel’ and ‘Pisang Awak’ had two unique sequences, ‘Zebrina GF’ had four and ‘Sukali Ndiizi’ had seven unique sequences (**Table 1**). All unique cDNA sequences from this study were deposited in GenBank (**Table 1**). The transcripts obtained were of variable lengths (471, 477, 504, 591, and 760 bp). The open reading frames of the cDNA sequences encoded proteins of about 156–167 amino acids. The *CENH3* sequence from ‘Calcutta 4’ (KT600803) was used as a reference as it had 100% identity to the exons of the publicly available genomic sequence of banana genotype ‘DH Pahang’ (GenBank accession number CAIC01023700 position 70772 to 76310). The protein translation of the ‘Calcutta 4’ cDNA sequence resulted in a 167 amino acid long protein.

**Table 1:** Description of *CENH3* transcripts from different genotypes of banana.

Genotype	Genomic group	Banana *CENH3* group	Unique sequence identifier	Total number of clones	CDS length	Exon/Intron Structure	Functional status	Genbank Accession Number
Gros Michel	AAA	*MusaCENH3-1B*	1	6	591	6/5	Non-functional	KP878227
Gros Michel	AAA	*MusaCENH3-1A*	2	7	504	7/6	Functional	KP878231
Pisang Awak	ABB	*MusaCENH3-1A*	4	5	504	7/6	Functional	KP878229
Pisang Awak	ABB	*MusaCENH3-1B*	5	4	760	5/4	Non-functional	KP878228
Sukali Ndiizi	AAB	*MusaCENH3-1B*	G	6	471	6/5	Functional	KP878221
Sukali Ndiizi	AAB	*MusaCENH3-1B*	A	4	504	7/6	Functional	KP878225
Sukali Ndiizi	AAB	*MusaCENH3-1B*	F	7	477	7/6	Functional	KP878222
Sukali Ndiizi	AAB	*MusaCENH3-1B*	H	3	504	7/6	Functional	KP878226
Sukali Ndiizi	AAB	*MusaCENH3-2*	B	5	471	5/4	Functional	KP878238
Sukali Ndiizi	AAB	*MusaCENH3-2*	C	4	471	5/4	Functional	KP878236
Sukali Ndiizi	AAB	*MusaCENH3-2*	E	4	471	5/4	Functional	KP878239
Zebrina GF	AA	*MusaCENH3-1B*	6	7	504	7/6	Functional	KP878223
Zebrina GF	AA	*MusaCENH3-1B*	7	9	504	7/6	Functional	KP878224
Zebrina GF	AA	*MusaCENH3-1B*	8	5	504	7/6	Functional	KP878220
Zebrina GF	AA	*MusaCENH3-2*	9	3	471	5/4	Functional	KP878237
*Musa balbisiana*	BB	*MusaCENH3-1A*	10	13	504	7/6	Functional	KT600804
Calcutta 4	AA	*MusaCENH3-1B*	11	13	504	7/6	Functional	KT600803


**FIGURE 1:**
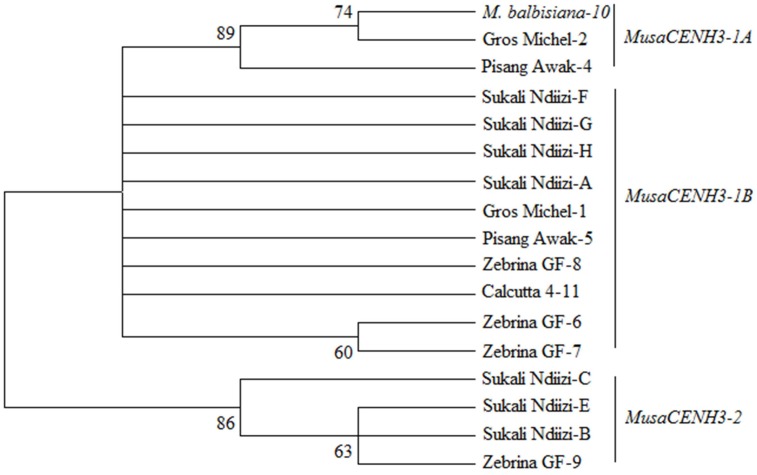
Phylogenetic tree of banana *CENH3s*. Unrooted Phylogenetic tree based on histone fold domain (HFD) and conserved *CENH3* tail sections of six banana genotypes showing the *MusaCENH3-1A, MusaCENH3-1B*, and *MusaCENH3-2* groups. Values at the root are bootstrap support values at 1000 replicates. The tree was drawn in MEGA 6 (Tamura et al., 2013).

**FIGURE 2:**
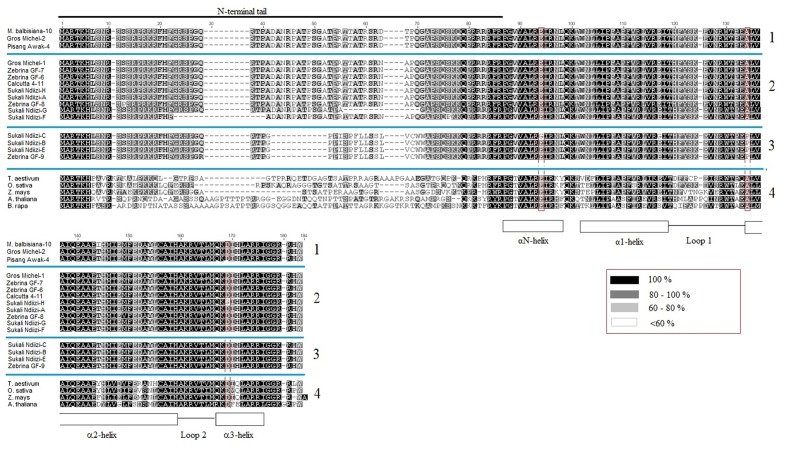
Multiple alignment of banana CENH3s. The blue lines separate the different alignments: Block 1 is a *MusaCENH3-1A* alignment, block 2 is a *MusaCENH3-1B*, block 3 is *MusaCENH3-2* and block 4 is an alignment to other monocots and dicots. The red highlights in the alignment are some amino acids substitutions observed in banana alleles within the HFD. Inset red box is the similarity index. Alignments were conducted in ClustalW (Larkin et al., 2007) as implemented in Geneious version 7.1 (Kearse et al., 2012).

**FIGURE 3:**
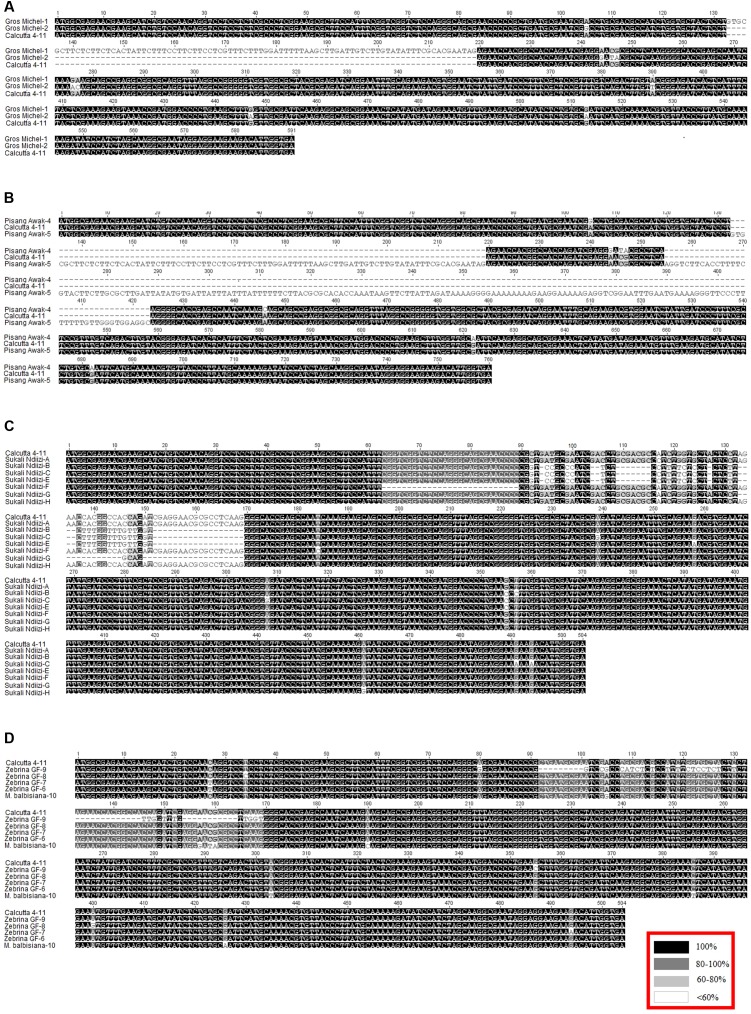
Alignment of transcript variants to the reference transcript from diploid banana genotype ‘Calcutta 4’. Blocks **(A–D)** are alignments of genotypes ‘Gros Michel’, ‘Pisang Awak’, ‘Sukali Ndiizi’, and a combination of ‘Zebrina GF’ and species ‘*Musa balbisiana*’ to ‘Calcutta 4’, respectively. Inset in red is the nucleotide alignment similarity index.

Based on similarity of conserved cDNA regions (partially in the tail and entire HFD region), the *CENH3* sequences were clustered into three major groups denoted as *MusaCENH3-1A* (transcripts of *M. balbisiana-10*, Gros Michel-2, Pisang Awak-4) *MusaCENH3-1B (*Gros Michel-1, Zebrina GF-6, 7, and 8, Calcutta 4-11, Pisang Awak-5, and Sukali Ndiizi-A, F, G and H) and *MusaCENH3-2* (Sukali Ndiizi-B, C and E and Zebrina GF-9) (**Figure 1** and **Table 1**). The transcripts within each group had slight variations mainly less than two SNPs. The first two groups (*MusaCENH3-1A* and *MusaCENH3-1B*) differed from *MusaCENH3-2* with a C to G substitution within the HFD α-2 helix region that resulted in alanine (A) to proline (P) substitution in the later. In addition to this HFD SNP, transcripts in *MusaCENH3-2* group consistently had a 46 bp longer exon 1 than *MusaCENH3-1A* and *MusaCENH3-1B* and also lacked extra two exons (exons 2 and 3), which were otherwise present in *MusaCENH3-1A* and *MusaCENH3-1B* groups. The 46 bp extra length in exon 1 as well as lack of exons 2 and 3 in *MusaCENH3-2* suggests that this is a different type of *CENH3* in bananas. However, since we did not sequence the whole genome of the genotypes used in this study, we cannot definitively prove that the missing exons or 46 bp extension are indeed different genes or splice variants (as suggested by alignment to the published sequence), but this seems likely and we will refer to them as such. There were also multiple SNPs within transcripts of each *CENH3* group, majority of which were within the HFD (**Figures 2**, **3**). In comparison to CENH3s from other monocots and dicot species, banana sequences were observed to be highly variable within the tail region and conserved only in the loop 2 of the HDF (**Figure 2**).

The *MusaCENH3-1A* and *MusaCENH3-1B* groups were more similar to each other in both sequence and splicing in comparison to transcripts in group *MusaCENH3-2*. The *MusaCENH3-1A* and *MusaCENH3-1B* transcripts differed at five SNP sites (**Figure 3**), which resulted in one non-synonymous amino acid substitution (**Figure 2**). The *MusaCENH3-1A* was observed in both A and B genomes. The genotypes ‘Zebrina GF’ and ‘Sukali Ndiizi’ had the highest number of SNP variants observed (**Table 2**).

**Table 2:** Minimum banana *CENH3* allele and splice variants.

Banana Genotype	Minimum number of SNP-allele variants	Minimum number of splice variants	Splicing mechanism (s)	Splice variant *CENH3* group(s)
Gros Michel	2	1	7/6, 6/5	*MusaCENH3-1A* and *-1B*
Pisang Awak	2	1	7/6, 5/4	*MusaCENH3-1A* and -*1B*
Sukali Ndiizi	4	6	7/6, 5/4, and 6/5	*MusaCENH3-1A, -1B* and *-2*
Zebrina GF	4	1	7/6 and 5/4	*MusaCENH3-1B* and *-2*
*Musa balbisiana*	1	0	7/6	*MusaCENH3-1A*
Calcutta 4	1	0	7/6	*MusaCENH3-1B*


These three banana *CENH3* groups differed in the number of exons as identified by alignment to the genomic sequence obtained through BLASTN analysis (CAIC01023700 position 70772–76310). The alignment confirmed that *MusaCENH3-1A* and *MusaCENH3-1B* have seven exons whereas *MusaCENH3-2* had five exons with exemptions of specific cases that differed due to exon skipping or intron retention. The *MusaCENH3-1A* and *MusaCENH3-1B* transcripts were 471 bp to 760 bp long while those in the *MusaCENH3-2* group were 471 bp long. The three *CENH3* groups had few SNPs among them that were observed mainly in transcripts from different genotypes.

To check the homology of banana CENH3 proteins to those of other plant species, banana translated protein sequences were aligned to monocot (*T. aestivum*, *O. sativa*, and *Z. mays*) and dicot species (*A. thaliana* and *B. rapa*). This alignment resulted in conserved αN-helix, α1-helix, α2-helix and α3-helix of the C-terminal, a specific loop 1 and a highly variable N-terminal tail (**Figure 2**). The loop 1 and α2-helix of the C-terminal constitute the CENP-A targeting domain (CATD) and these two domains were found to be conserved in banana sequences except for two amino acid substitutions within the α2-helix in the sequences *M. balbisiana-10* (alignment position 144) and Sukali Ndiizi-B, C, and E and in the Zebrina GF-9 (alignment position 134) (**Figure 2**).

### Variants in Autotriploid Genotype ‘Gros Michel’

‘Gros Michel’ had two variable and unique sequences as grouped in *MusaCENH3-1A* (Gros Michel-2) and *MusaCENH3-1B* (Gros Michel-1). Over and above having the five SNPs that differentiated *MusaCENH3-1A* from *MusaCENH3-1B*, the transcript Gros Michel-1 had an 87 bp indel that resulted from retention of intron 2 and spanned alignment positions 133 to 219 (**Figure 3A**). This retained intron resulted in a frame shift and introduced a premature stop codon in the tail region (nucleotide position 219) rendering it non-functional. The transcript Gros Michel-2 differed to Calcutta 4 at nine SNPs and out of these, six were in the tail region. Two (alignment positions 105 and 276) out of six SNPs in the tail were synonymous substitutions. The other four SNPs were aligned at positions 244, 246, 247, and 277 resulted in a total of three amino acid substitutions.

### Variants in Allotriploid Genotype ‘Pisang Awak’

The allotriploid cultivated genotype ‘Pisang Awak’ had two unique transcripts that fell into the *CENH3* groups *MusaCENH3-1A* (Pisang Awak-5) and *MusaCENH3-1B* (Pisang Awak-4). Despite being in the *MusaCENH3-1B* group, the transcript Pisang Awak-5 had retained two introns (introns 2 and 3) (**Figure 3B**). These retained introns resulted in a non-functional protein by introducing multiple premature stop codons the first one at nucleotide position 219 in the tail region. The transcript Pisang Awak-4 carried two additional SNPs [one in the tail and one in HFD (alignment positions 105 and 622)] in addition to the five that allowed it to be grouped into the *MusaCENH3-1B.* Both SNPs were silent and did not result in any amino acid substitution.

### Variants in Allotriploid Genotype ‘Sukali Ndiizi’

The allotriploid genotype ‘Sukali Ndiizi’ had seven variants, four within *MusaCENH3-1B* group (transcripts Sukali Ndiizi-A, F, G, and H) and three within *MusaCENH3-2* (Sukali Ndiizi-B, C, and E). Despite being in the same *MusaCENH3-1B* group, the sequences of Sukali Ndiizi-A and H differed to the Calcutta 4 at one SNP position each; positions 117 (A to C) and 461 (A to G) in Sukali Ndiizi-A and H, respectively, with the latter resulting in a aspartic acid (D) to glycine (G) substitution in the protein sequence (**Figures 2**, **3C**). The transcripts Sukali Ndiizi-F and G varied from each other with indels; Sukali Ndiizi-F had 27 bp indel (alignment position 63 – 89) as well as substitution from T to C at position 184 which resulted in a serine (S) to proline (P) substitution in the protein translation. The 27 bp indel resulted in a shortened protein sequence with 158 amino acids due to a deletion in exon 1. The transcript Sukali Ndiizi-G on the other hand had a 37 bp indel from alignment positions 133–169 (from skipping of exon 3), which resulted in alternative 3′ and 5′ splice sites. The two splice variations did not cause any shift in the reading frames and therefore resulted in functional proteins.

The transcripts falling within the *MusaCENH3-2* group (Sukali Ndiizi-C, B, and E) were observed to be 471 bp long, which is 33 bp shorter than those in *MusaCENH3-1A* and *MusaCENH3-1B* and especially with Calcutta 4 (**Figure 3C**). The resultant proteins were all 156 amino acids long and functional. Despite all the three transcripts being in the same group (*MusaCENH3-2*) they differed among themselves at six nucleotide positions, four of which resulted in amino acid substitutions at positions 94 and 117, 179 and 181 in Sukali Ndiizi-C (**Figure 2**).

### Variants in Diploid Banana ‘Zebrina GF’ and ‘*Musa balbisiana*’

The diploid banana genotype ‘Zebrina GF’ expressed transcripts that fell into both the *MusaCENH3-1B* (transcripts Zebrina GF-6, 7, and 8) and *MusaCENH3-2* (Zebrina GF-9) categories. The three transcripts in *MusaCENH3-1B* differed amongst themselves with four SNPs, three of which were non-synonymous substitutions at alignment positions 34 (T to C) and 400 (A to G) in Zebrina GF-8 and position 494 in Zebrina GF-7 (G to A) (**Figure 3D**). The only *MusaCENH3-2* representative sequence in this genotype was transcript Zebrina GF-9, which was 471 bp encoding a 156 amino acid long protein. This transcript, like others in the same group from other cultivars, had an exon 1 that was 45 bp longer, the C to G substitution in the α-2 helix and in addition an A to G non-synonymous substitution at alignment position 80 (**Figure 3D**) that resulted in glycine (Q) to argenine (R) substitution at protein alignment position 28 (**Figure 2**).

The diploid species ‘*M. balbisiana’* had one unique 504 bp long sequence that encoded a 167 amino acid long protein. This transcript fell into the *MusaCENH3-1A* group and differed from others in the same group with one major non-synonymous SNP site in the HFD that resulted in the substitution of the amino acid threonine (T) to isoleucine (I) at alignment position 144.

### Exon/Intron Structures in Banana *CENH3*

**FIGURE 4:**
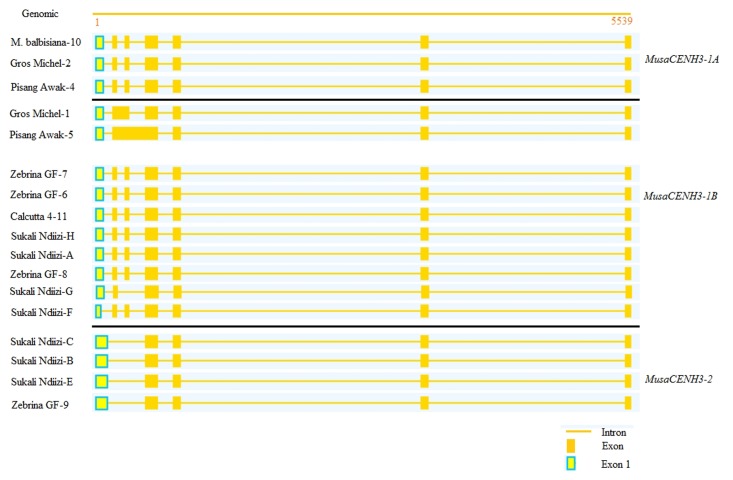
Alignment of the *CENH3* transcripts to genomic sequence of genotype ‘DH Pahang’ to identify splice mechanisms. The 7/6, 6/5, and 5/4 structures are represented intron/exon structures are represented. Alignment was performed using Splign (Kapustin et al., 2008).

To get an insight into the splicing approaches and the intron/exon structures of the transcripts obtained and to also know if the differences in lengths of the transcripts were due to splicing variations, the unique banana *CENH3* transcripts were mapped to genomic sequence of putative *CENH3* from ‘DH Pahang’ (**Figure 4** and **Additional File S2**). Three exon/intron structures (7/6, 6/5, and 5/4) were observed, which were probably as a result of differences in splicing patterns (**Figure 4**). The 7 exon/6 intron structure was most frequently observed (10 transcripts out of 17 unique clones). This structure was observed in both diploid and triploid genotypes with three of the four transcripts from the diploid ‘Zebrina GF’ (Zebrina GF-6, 7, and 8), diploid ‘Calcutta 4’ and ‘*M. balbisiana’*, triploid genotype ‘Pisang Awak’ (Pisang Awak-4), in three of the seven sequences in the genotype ‘Sukali Ndiizi’ (Sukali Ndiizi-A, F, and H) and in one transcript from the autopolyploid ‘Gros Michel’ (Gros Michel-2) (**Table 1**).

The 5 exon/4 intron structure was observed in genotypes ‘Pisang Awak’ (Pisang Awak-5), ‘Sukali Ndiizi’ (Sukali Ndiizi-B, C, and E) and the diploid ‘Zebrina GF’ (Zebrina GF-9) (**Figure 4**). This structure resulted from skipping of the second and the third exons in all sequences apart from Pisang Awak-5, which had this structure due to retention of introns two and three.

The 6 exon/5 intron pattern was observed in two transcripts of Gros Michel-1 and Sukali Ndiizi-G from the genotype ‘Gros Michel’ and ‘Sukali Ndiizi’, respectively (**Figure 4**). This structure was as a result of skipping of exon 2 for Sukali Ndiizi-G and retention of intron 2 in the transcript Gros Michel-1.

### Alternative Splicing of *CENH3* in Banana

Alternative splicing achieves diversity and novelty of proteins. Alternatively spliced variants were obtained based on deviations from splicing in their respective banana *CENH3* groups. Four out of the seventeen unique transcripts were alternatively spliced with two of these resulting in unique proteins while the rest introduced premature stop codons. Some of the alternatively spliced transcripts also had SNP variations. Three alternative splicing approaches were observed: exon skipping, intron retention and alternate 3′ and 5′ splice site (**Figure 4** and **Additional File S2**).

### Alternative Splicing by Exon Skipping

Exon skipping was observed in only one transcript (Sukali Ndiizi-G) of the triploid cultivar ‘Sukali Ndiizi’. This alternate splicing mechanism resulted from skipping of exon 3 and resulted in a functional transcript (**Figure 4** and **Additional File S2**). Exon skipping resulted in shorter transcript length, where the transcript affected (Sukali Ndiizi-G) had a reduced length of 471 bp instead of the 504 bp in transcripts from the same *CENH3* group.

### Alternative Splicing by Intron Retention

Intron retention as an alternative splicing mechanism was observed in two transcripts (Gros Michel-1 and Pisang Awak-5), which retained one and two introns, respectively (**Figure 4**). The intron retention resulted in non-functional proteins due to introduction of at least one stop codon in either of the two transcripts. The transcript Pisang Awak-5 had five stop codons introduced, four in the tail and one in the HFD, whereas Gros Michel-1 only had one stop codon in the tail region.

### Splice Variation by Alternative Splice Site Selection

The alternate 3′ and 5′ splice site selection resulted in variation in the length of exon 1 (**Figure 4**). Partial deletion of a 27 bp segment from positions 63–89 of exon 1 was observed in Sukali Ndiizi-F. This deletion resulted in a change of the splice junction from CCCC/GGTC to TTTC/GGTC resulting in a change in splice sites. The transcript Sukali Ndiizi-G was also observed to have a different splice site selection by retaining the nucleotide G from intron 2 (Exon 3 was skipped) and retaining the nucleotide C of intron 3.

### Secondary Structure Prediction

**FIGURE 5:**
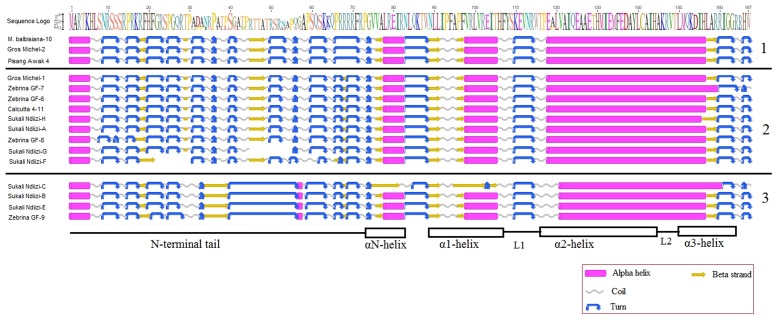
Predicted secondary structures of banana CENH3. 1- *MusaCENH3-1A*, 2*- MusaCENH3-1B*, and 3- *MusaCENH3-2.* At the bottom are the different CENH3 domains, above the structures are the amino acids in logo format. Inset is the key to the secondary structures.

There was general conservation in predicted secondary structure within each of the banana *CENH3* groups, although slight variations at specific sections were observed (**Figure 5**). The *MusaCENH3-1A* and *MusaCENH3-1B* had similar predicted secondary folding and varied in the second and the third last turns of the N-terminal tail where they were merged into one due to the lack of the predicted intervening beta sheet in the *MusaCENH3-1A* group. The secondary structures in the *MusaCENH3-2* had more structural variation in comparison to the *MusaCENH3-1A* and *MusaCENH3-1B*. The structural modifications included addition, loss, elongation or shortening of coils, turns, α-helices and β-sheets. Splice variations affecting the tail region resulted in loss of α-helices and beta strands, coils and turns in Sukali Ndiizi-G and F (**Figure 5**). The CENH3 proteins for Sukali Ndiizi-C, B, E, and Zebrina GF-9 also gained and lost domains within the tail region. The major form of variation observed within the HFD was point mutations some of which resulted in non-synonymous substitution. These substitutions resulted in elongations and/or shortening of some predicted secondary structures. The Proline (P) to Alanine (A) substitution within the HFD in Sukali Ndiizi-C, B, E, and Zebrina GF-9 resulted in an elongated loop 1 and a shortened α2 helix a clear structural feature unique to the *MusaCENH3-2* CENH3 groups (**Figure 5**). The substitutions of glutamine (E) with glycine (G) at two different positions in Sukali Ndiizi-C resulted in structural changes within the αN- and α1-helices (**Figure 5**).

## Discussion

In this study, we observed that *CENH3* in diploid and triploid bananas exists as single or multiple allele variants depending on the genotype. The variants were SNPs in both the tail and the HFD region of CENH3. The non-synonymous SNPs resulted in modification of the predicted secondary structures of proteins. The majority of splice variants (apart from two) were predicted to translate in-frame. These splice variations only affected the tail region of CENH3. The presence of multiple *CENH3* SNP-alleles in a diploid genotype like ‘Zebrina GF’ suggests that bananas maybe carrying more than one *CENH3* gene per genome.

Cultivated bananas are mainly triploids. Banana breeding involves crossing fertile triploids with diploids to get tetraploids which are then crossed to diploid accessions to give triploid cultivars (Pillay et al., 2004). In other plant species, the presence of different *CENH3s* from different parents in embryos has been observed to result in uniparental genome elimination, aneuploids, or stable hybrids (Ravi and Chan, 2010; Kuppu et al., 2015; Maheshwari et al., 2015; Tan et al., 2015; Kelliher et al., 2016). The focus in banana breeding programs is to first establish tetraploids and then use them to develop triploids. It has been suggested that crosses between diploids and triploids result in viable diploids (De Langhe et al., 2010). Crosses between *A. thaliana* wild-type parents and pollen donors carrying specific point mutations within the HFD resulted in uniparental genome elimination- with loss of the genome derived from the mutant line (Kuppu et al., 2015). Two out of the five point mutations that resulted in uniparental genome elimination in Arabidopsis were within the centromere targeting domain (CATD). Some of the SNPs in our study were observed to be within the conserved HFD domains including α2- and α3-helices. Furthermore, mutations of CENP-A (CENH3 of humans) residues resulted in reduced retention of CENP-A in centromere of human cells and this was due to the effect on α2-helix length which plays a key role of maintaining orientation at nucleosome entry and exit (Tachiwana et al., 2011). The SNPs were within the CATD and these resulted in a predicted shortening of the α2-helix. These CATD SNP variations can result in CENH3 nucleosome instability and may affect crosses of bananas having *CENH3s* with variation at these positions. It would be interesting to know if multiple *CENH3s* affect banana breeding and if they do, then it may be important to consider *CENH3* type when choosing parents for crossing.

We observed three *CENH3* variants in both diploid and triploid bananas, which differed at the tail region and have SNPs in the HFD region. The presence of these three variants in a diploid line indicates presence of more than one *CENH3* in a single banana genome. Alpha and beta *CENH3* variants in wheat were observed to have different functional roles. Reduced expression of alpha version resulted in extreme dwarfing and weakened root system whereas reduced expression of the beta version resulted in reduced plant height and reproductive fitness leading to the conclusion that the two versions are involved in plant development and reproductive development, respectively (Yuan et al., 2015). Although this study did not explore the functions of the banana *CENH3* variants, it would be worth conducting such studies in future to verify if the variants differ in functionality.

The presence of multiple *CENH3* allele variants in a wild diploid banana (four in diploid ‘Zebrina GF’) corroborate the hypothesis that domestication of cultivated hybrids passed through intermediate hybrids (De Langhe et al., 2010). ‘Zebrina GF’ is a wild diploid that has been shown to segregate during crosses, an indication that it has a high degree of heterozygozity (personal communication from Professor Rony Swennen, Banana breeder at IITA and collector of this genotype).

The observation that alternative splicing of *CENH3* in bananas only affected the N-terminal tail is consistent with those made in the angiosperms *Oryza* spp., *Brassica* spp., and *Gossypium* spp. (Wang et al., 2011; Masonbrink et al., 2014). It was interesting to observe that some of the splice variations resulted in transcripts that were translatable into proteins as these could further add into the diversity of banana *CENH3s.* However, it is not clear if the in-frame splice variants translate into proteins *in vivo* and whether they are loaded into the centromere. The role of the out-of-frame variants is also not clear and future studies targeting the *CENH3* splice variants and their proteins (if translated) are required to identify their role(s) and fate.

The variations observed in the three main banana *CENH3* groups were observed to affect the predicted secondary structures of the respective proteins. This is interesting considering that crosses of Arabidopsis null mutant lines complemented with a *CENH3* version in which tail was replaced with that of histone H3.3 and GFP-tagged to wild-type resulted in uniparental genome elimination (Ravi and Chan, 2010). The highly variable N-terminal tail of the *CENH3* indicates its role in the evolving centromeric satellites (Hui et al., 2010; Ravi et al., 2010; Hayden and Willard, 2012) or affecting the targeting of centromeres that might be a mode of bringing in new CENH3 proteins in response to increased centromere size (Masonbrink et al., 2014).

The frequency of non-synonymous SNPs within each of the banana *CENH3* groups was observed to be higher within the HFD region, while the frequency of both synonymous and non-synonymous SNPs between different *CENH3* groups was higher in the tail region. A study on evolution of *CENH3* in drosophila observed that the frequency of interspecific *CENH3* polymorphisms were higher in the tail than the HFD although the ratios of such changes were lower within the same species (Malik and Henikoff, 2001). One of the *CENH3* groups (*MusaCENH3-1A*) was observed to be specific to the diploid *Musa* species ‘*M. balbisiana*’ and differed from other *CENH3* groups with non-synonymous substitutions, majority of which were in the tail region. Majority of the non-synonymous SNPs in transcripts within banana *CENH3* group were observed to be within the CATD, which may affect CENH3 targeting to the centromere because the CATD specifically the loop 1 has been shown to be involved in localization (Dalal et al., 2007).

The number of *CENH3* exons and introns in the respective exon/intron structures has been found to vary in different plant species. Seven *CENH3* transcripts obtained from five rice species were observed to have 7 exons and 6 introns despite having different CDS lengths (Hirsch et al., 2009). In carrots, a similar structure of 7 exons and 6 introns was observed while in brassica two different structures were observed in *CENH3s* of varying lengths, one with 7 exons/6 introns and second with 9 exons/8 introns structure. In this study, three exons/introns structures (7/6, 6/5, and 5/4) were observed in bananas. The 7/6 and the 5/4 exon/intron structures were found in both diploids and triploids, however, 7/6 was most predominant. The 6 exons/5 introns structure was only observed in triploid bananas and this mechanism resulted in functional and non-functional transcripts. In this analysis, it is clear that there was more bias toward having a 7 exons/6 introns structure whereas the 5 exons/4 introns structure was minor and the 6/5 structure was a result of failure of the 7/6 to splice correctly.

This study provided insight into how *CENH3* is expressed in diploid and triploid bananas. Additional genotypes including tetraploids should be included in future studies. Due to the emergence of CENH3-based breeding techniques, the knowledge obtained here indicates that checking the *CENH3* type may be used as a criterion in selection of parents for banana breeding.

## Availability of Supporting Data

Gene sequences for banana *CENH3* obtained in this study were deposited in the GenBank and the accession numbers are provided in this manuscript, all other supporting data is providing as additional files.

## Author Contributions

KM, LT, AB, and SC conceived the idea and designed the experiments. KM performed the experiments and wrote the manuscript. LT and AB supervised the experimentation. All authors contributed in interpreting the data. KM, AB, LT, NA, and EN contributed to reviewing and editing the manuscript.

## Conflict of Interest Statement

The authors declare that the research was conducted in the absence of any commercial or financial relationships that could be construed as a potential conflict of interest.

## Supplementary Material

**ADDITIONAL FILE S1 |** BLAST hits on *Musa acuminata* whole genome shot gun contigs using *A. thaliana CENH3* genomic sequence as the query.

**ADDITIONAL FILE S2 |** Alignment of banana *CENH3* transcripts to the putative genomic sequence obtained using *A. thaliana CENH3* genomic sequence to query *M. acuminata* whole genome contig.
