# Supplementary material for: Expressed Centromere Specific Histone 3 (CENH3) Variants in Cultivated Triploid and Wild Diploid Bananas (Musa spp.)
Source: Front Plant Sci. 2017 Jun 29;8:1034. doi: 10.3389/fpls.2017.01034 (PMC5489561; doi:10.3389/fpls.2017.01034)
Supplement: Supplementary file 1 [file Data_Sheet_1.PDF]

BLASTN 2.2.32+
Reference: Stephen F. Altschul, Thomas L. Madden, Alejandro A. Schaffer, Jinghui Zhang, Zheng Zhang, Webb Miller, and David J. Lipman (1997), "Gapped BLAST and PSI-BLAST: a new generation of protein database search programs", Nucleic Acids Res. 25:3389-3402.

RID: Y81PB3ME015

Database: WGS_VDB://CAIC01
24,424 sequences; 390,578,572 total letters
Query=
Length=2087

Sequences producing significant alignments:		Score (Bits)	E Value
emb|CAIC01023614.1|	Musa acuminata subsp. malaccensis WGS pro...	48.2	0.003
emb|CAIC01023700.1|	Musa acuminata subsp. malaccensis WGS pro...	46.4	0.009
emb|CAIC01013510.1|	Musa acuminata subsp. malaccensis WGS pro...	46.4	0.009
emb|CAIC01017372.1|	Musa acuminata subsp. malaccensis WGS pro...	44.6	0.031
emb|CAIC01010666.1|	Musa acuminata subsp. malaccensis WGS pro...	44.6	0.031
emb|CAIC01008443.1|	Musa acuminata subsp. malaccensis WGS pro...	44.6	0.031
emb|CAIC01023792.1|	Musa acuminata subsp. malaccensis WGS pro...	41.0	0.37
emb|CAIC01018285.1|	Musa acuminata subsp. malaccensis WGS pro...	41.0	0.37
emb|CAIC01018123.1|	Musa acuminata subsp. malaccensis WGS pro...	41.0	0.37
emb|CAIC01014880.1|	Musa acuminata subsp. malaccensis WGS pro...	41.0	0.37
emb|CAIC01014332.1|	Musa acuminata subsp. malaccensis WGS pro...	41.0	0.37
emb|CAIC01013944.1|	Musa acuminata subsp. malaccensis WGS pro...	41.0	0.37
emb|CAIC01011835.1|	Musa acuminata subsp. malaccensis WGS pro...	41.0	0.37
emb|CAIC01010731.1|	Musa acuminata subsp. malaccensis WGS pro...	41.0	0.37
emb|CAIC01010505.1|	Musa acuminata subsp. malaccensis WGS pro...	41.0	0.37
emb|CAIC01010491.1|	Musa acuminata subsp. malaccensis WGS pro...	41.0	0.37
emb|CAIC01010108.1|	Musa acuminata subsp. malaccensis WGS pro...	41.0	0.37
emb|CAIC01008433.1|	Musa acuminata subsp. malaccensis WGS pro...	41.0	0.37
emb|CAIC01008158.1|	Musa acuminata subsp. malaccensis WGS pro...	41.0	0.37
emb|CAIC01007804.1|	Musa acuminata subsp. malaccensis WGS pro...	41.0	0.37
emb|CAIC01005535.1|	Musa acuminata subsp. malaccensis WGS pro...	41.0	0.37
emb|CAIC01017495.1|	Musa acuminata subsp. malaccensis WGS pro...	39.2	1.3
emb|CAIC01016426.1|	Musa acuminata subsp. malaccensis WGS pro...	39.2	1.3
emb|CAIC01012475.1|	Musa acuminata subsp. malaccensis WGS pro...	39.2	1.3
emb|CAIC01024381.1|	Musa acuminata subsp. malaccensis WGS pro...	37.4	4.6
emb|CAIC01024256.1|	Musa acuminata subsp. malaccensis WGS pro...	37.4	4.6
emb|CAIC01023384.1|	Musa acuminata subsp. malaccensis WGS pro...	37.4	4.6
emb|CAIC01023101.1|	Musa acuminata subsp. malaccensis WGS pro...	37.4	4.6
emb|CAIC01022935.1|	Musa acuminata subsp. malaccensis WGS pro...	37.4	4.6
emb|CAIC01022901.1|	Musa acuminata subsp. malaccensis WGS pro...	37.4	4.6
emb|CAIC01022550.1|	Musa acuminata subsp. malaccensis WGS pro...	37.4	4.6
emb|CAIC01022177.1|	Musa acuminata subsp. malaccensis WGS pro...	37.4	4.6
emb|CAIC01021950.1|	Musa acuminata subsp. malaccensis WGS pro...	37.4	4.6
emb|CAIC01021109.1|	Musa acuminata subsp. malaccensis WGS pro...	37.4	4.6
emb|CAIC01020790.1|	Musa acuminata subsp. malaccensis WGS pro...	37.4	4.6

```
emb|CAIC01019226.1|  Musa acuminata subsp. malaccensis WGS pro...  37.4    4.6
emb|CAIC01018551.1|  Musa acuminata subsp. malaccensis WGS pro...  37.4    4.6
emb|CAIC01018434.1|  Musa acuminata subsp. malaccensis WGS pro...  37.4    4.6
emb|CAIC01015797.1|  Musa acuminata subsp. malaccensis WGS pro...  37.4    4.6
emb|CAIC01014197.1|  Musa acuminata subsp. malaccensis WGS pro...  37.4    4.6
emb|CAIC01013428.1|  Musa acuminata subsp. malaccensis WGS pro...  37.4    4.6
emb|CAIC01012759.1|  Musa acuminata subsp. malaccensis WGS pro...  37.4    4.6
emb|CAIC01011690.1|  Musa acuminata subsp. malaccensis WGS pro...  37.4    4.6
emb|CAIC01010724.1|  Musa acuminata subsp. malaccensis WGS pro...  37.4    4.6
emb|CAIC01009744.1|  Musa acuminata subsp. malaccensis WGS pro...  37.4    4.6
emb|CAIC01008714.1|  Musa acuminata subsp. malaccensis WGS pro...  37.4    4.6

ALIGNMENTS
>emb|CAIC01023614.1| Musa acuminata subsp. malaccensis WGS project CAIC00000000
data,
strain Doubled-haploid Pahang (DH-Pahang), contig_811, whole
genome shotgun sequence
Length=308979

 Score = 48.2 bits (52),  Expect = 0.003
 Identities = 51/65 (78%), Gaps = 7/65 (11%)
 Strand=Plus/Plus

Query  572     ttttttC-CCTCTTTTCATCT-CTTTTGTTTGTGAAGTTATTCTTTTGTAACATCTGCAG  629
               || |||| |||||||| | || ||||||||||| |||| ||||| |     |||||||||
Sbjct  161142  TTGTTTCACCTCTTTTTAGCTTCTTTTGTTTGTTAAGTGATTCTCT-----CATCTGCAG
161196

Query  630     CAAAT  634
               || ||
Sbjct  161197  CACAT  161201


>emb|CAIC01023700.1| Musa acuminata subsp. malaccensis WGS project CAIC00000000
data,
strain Doubled-haploid Pahang (DH-Pahang), contig_725, whole
genome shotgun sequence
Length=82928

 Score = 46.4 bits (50),  Expect = 0.009
 Identities = 42/53 (79%), Gaps = 0/53 (0%)
 Strand=Plus/Plus

Query  1403   AATCGTTGGACAGCTGAAGCTCTTGTTGCTCTTCAAGAGGTACCAATCCTTCA  1455
              || || |||||  |||||||| | |||||  ||||||||||||   |||||||
Sbjct  71619  AACCGATGGACCCCTGAAGCTTTGGTTGCGATTCAAGAGGTACTTTTCCTTCA  71671


>emb|CAIC01013510.1| Musa acuminata subsp. malaccensis WGS project CAIC00000000
data,
strain Doubled-haploid Pahang (DH-Pahang), contig_10916,
whole genome shotgun sequence
Length=40370

 Score = 46.4 bits (50),  Expect = 0.009
```

```
 Identities = 36/42 (86%), Gaps = 1/42 (2%)
 Strand=Plus/Plus

Query  1714   TTGAATCATTAGTTACTTGAATATCATATATAAAATGCTTAT  1755
              ||| ||||| | |  |||||||||||||||||||||||| ||
Sbjct  39582  TTGTATCATGAATG-CTTGAATATCATATATAAAATGCTCAT  39622


>emb|CAIC01017372.1| Musa acuminata subsp. malaccensis WGS project CAIC00000000
data,
strain Doubled-haploid Pahang (DH-Pahang), contig_7054,
whole genome shotgun sequence
Length=12150

 Score = 44.6 bits (48),  Expect = 0.031
 Identities = 27/29 (93%), Gaps = 0/29 (0%)
 Strand=Plus/Minus

Query  1727  TACTTGAATATCATATATAAAATGCTTAT  1755
             ||||||||||||||||||| ||||| ||
Sbjct  3669  TACTTGAATATCATATATAATATGCTCAT  3641


>emb|CAIC01010666.1| Musa acuminata subsp. malaccensis WGS project CAIC00000000
data,
strain Doubled-haploid Pahang (DH-Pahang), contig_13760,
whole genome shotgun sequence
Length=31469

 Score = 44.6 bits (48),  Expect = 0.031
 Identities = 27/29 (93%), Gaps = 0/29 (0%)
 Strand=Plus/Minus

Query  1727   TACTTGAATATCATATATAAAATGCTTAT  1755
              ||||||||||||||||||| ||||| ||
Sbjct  15395  TACTTGAATATCATATATAATATGCTCAT  15367


>emb|CAIC01008443.1| Musa acuminata subsp. malaccensis WGS project CAIC00000000
data,
strain Doubled-haploid Pahang (DH-Pahang), contig_15983,
whole genome shotgun sequence
Length=6169

 Score = 44.6 bits (48),  Expect = 0.031
 Identities = 27/29 (93%), Gaps = 0/29 (0%)
 Strand=Plus/Plus

Query  1727  TACTTGAATATCATATATAAAATGCTTAT  1755
             ||||||||||||||||||| ||||| ||
Sbjct  6044  TACTTGAATATCATATATAATATGCTCAT  6072


>emb|CAIC01023792.1| Musa acuminata subsp. malaccensis WGS project CAIC00000000
data,
```

```
strain Doubled-haploid Pahang (DH-Pahang), contig_633, whole
genome shotgun sequence
Length=63539

 Score = 41.0 bits (44),  Expect = 0.37
 Identities = 28/32 (88%), Gaps = 0/32 (0%)
 Strand=Plus/Plus

Query  571    tttttttCCCTCTTTTCATCTCTTTTGTTTGT  602
              ||||||| ||| || ||||||||||| |||||
Sbjct  48063  TTTTTTTTCCTTTTCTCATCTCTTTTATTTGT  48094


>emb|CAIC01018285.1| Musa acuminata subsp. malaccensis WGS project CAIC00000000
data,
strain Doubled-haploid Pahang (DH-Pahang), contig_6141,
whole genome shotgun sequence
Length=46660

 Score = 41.0 bits (44),  Expect = 0.37
 Identities = 35/41 (85%), Gaps = 2/41 (5%)
 Strand=Plus/Plus

Query  1730  TTGAATA-TCATATATAAAATGCTTATCTATATCTGTTTTT  1769
             ||||||| ||||||  ||||||||||||| ||| | |||||
Sbjct  8468  TTGAATAGTCATATTGAAAATGCTTATCT-TATTTCTTTTT  8507


>emb|CAIC01018123.1| Musa acuminata subsp. malaccensis WGS project CAIC00000000
data,
strain Doubled-haploid Pahang (DH-Pahang), contig_6303,
whole genome shotgun sequence
Length=16081

 Score = 41.0 bits (44),  Expect = 0.37
 Identities = 28/32 (88%), Gaps = 0/32 (0%)
 Strand=Plus/Plus

Query  1769  TTGTTCATATCAGTGAGAAAAGACTTTGAACT  1800
             ||| |||||||||||||||  ||||| |||||
Sbjct  5403  TTGGTCATATCAGTGAGAAGGGACTTCGAACT  5434


>emb|CAIC01014880.1| Musa acuminata subsp. malaccensis WGS project CAIC00000000
data,
strain Doubled-haploid Pahang (DH-Pahang), contig_9546,
whole genome shotgun sequence
Length=23971

 Score = 41.0 bits (44),  Expect = 0.37
 Identities = 25/27 (93%), Gaps = 0/27 (0%)
 Strand=Plus/Plus

Query  1729   CTTGAATATCATATATAAAATGCTTAT  1755
              |||||||||||||||||| ||||| ||
```

```
Sbjct  17751  CTTGAATATCATATATAATATGCTCAT  17777


>emb|CAIC01014332.1| Musa acuminata subsp. malaccensis WGS project CAIC00000000
data,
strain Doubled-haploid Pahang (DH-Pahang), contig_10094,
whole genome shotgun sequence
Length=37012

 Score = 41.0 bits (44),  Expect = 0.37
 Identities = 25/27 (93%), Gaps = 0/27 (0%)
 Strand=Plus/Minus

Query  1729   CTTGAATATCATATATAAAATGCTTAT  1755
              ||||||||||||||||| ||||| ||
Sbjct  29135  CTTGAATATCATATATAATATGCTCAT  29109


>emb|CAIC01013944.1| Musa acuminata subsp. malaccensis WGS project CAIC00000000
data,
strain Doubled-haploid Pahang (DH-Pahang), contig_10482,
whole genome shotgun sequence
Length=36033

 Score = 41.0 bits (44),  Expect = 0.37
 Identities = 28/32 (88%), Gaps = 0/32 (0%)
 Strand=Plus/Plus

Query  1769   TTGTTCATATCAGTGAGAAAAGACTTTGAACT  1800
              ||| |||||||||||||| ||||||  ||||
Sbjct  31774  TTGGTCATATCAGTGAGAAGAGACTTCAAACT  31805


>emb|CAIC01011835.1| Musa acuminata subsp. malaccensis WGS project CAIC00000000
data,
strain Doubled-haploid Pahang (DH-Pahang), contig_12591,
whole genome shotgun sequence
Length=18157

 Score = 41.0 bits (44),  Expect = 0.37
 Identities = 25/27 (93%), Gaps = 0/27 (0%)
 Strand=Plus/Plus

Query  1729  CTTGAATATCATATATAAAATGCTTAT  1755
             ||||||||||||||||| ||||| ||
Sbjct  8918  CTTGAATATCATATATAATATGCTCAT  8944


>emb|CAIC01010731.1| Musa acuminata subsp. malaccensis WGS project CAIC00000000
data,
strain Doubled-haploid Pahang (DH-Pahang), contig_13695,
whole genome shotgun sequence
Length=39344

 Score = 41.0 bits (44),  Expect = 0.37
```

Identities = 25/27 (93%), Gaps = 0/27 (0%)
Strand=Plus/Minus

```
Query  1729  CTTGAATATCATATATAAAATGCTTAT  1755
             ||||||||||||||||||| ||||| ||
Sbjct  5530  CTTGAATATCATATATAATATGCTCAT  5504
```

>emb|CAIC01010505.1| Musa acuminata subsp. malaccensis WGS project CAIC00000000 data,
strain Doubled-haploid Pahang (DH-Pahang), contig_13921,
whole genome shotgun sequence
Length=50640

Score = 41.0 bits (44), Expect = 0.37
Identities = 25/27 (93%), Gaps = 0/27 (0%)
Strand=Plus/Minus

```
Query  1729   CTTGAATATCATATATAAAATGCTTAT  1755
              ||||||||||||||||||| ||||| ||
Sbjct  16715  CTTGAATATCATATATAATATGCTCAT  16689
```

>emb|CAIC01010491.1| Musa acuminata subsp. malaccensis WGS project CAIC00000000 data,
strain Doubled-haploid Pahang (DH-Pahang), contig_13935,
whole genome shotgun sequence
Length=6043

Score = 41.0 bits (44), Expect = 0.37
Identities = 25/27 (93%), Gaps = 0/27 (0%)
Strand=Plus/Plus

```
Query  1729  CTTGAATATCATATATAAAATGCTTAT  1755
             ||||||||||||||||||| ||||| ||
Sbjct  3176  CTTGAATATCATATATAATATGCTCAT  3202
```

>emb|CAIC01010108.1| Musa acuminata subsp. malaccensis WGS project CAIC00000000 data,
strain Doubled-haploid Pahang (DH-Pahang), contig_14318,
whole genome shotgun sequence
Length=1957

Score = 41.0 bits (44), Expect = 0.37
Identities = 25/27 (93%), Gaps = 0/27 (0%)
Strand=Plus/Minus

```
Query  1729  CTTGAATATCATATATAAAATGCTTAT  1755
             ||||||||||||||||||| ||||| ||
Sbjct  691   CTTGAATATCATATATAATATGCTCAT  665
```

>emb|CAIC01008433.1| Musa acuminata subsp. malaccensis WGS project CAIC00000000 data,

```
strain Doubled-haploid Pahang (DH-Pahang), contig_15993,
whole genome shotgun sequence
Length=2873

 Score = 41.0 bits (44),  Expect = 0.37
 Identities = 28/32 (88%), Gaps = 0/32 (0%)
 Strand=Plus/Plus

Query  1769  TTGTTCATATCAGTGAGAAAAGACTTTGAACT  1800
              ||| |||||||||||||||| |||||  ||||
Sbjct  1017  TTGGTCATATCAGTGAGAAAGGACTTCAAACT  1048


>emb|CAIC01008158.1| Musa acuminata subsp. malaccensis WGS project CAIC00000000
data,
strain Doubled-haploid Pahang (DH-Pahang), contig_16268,
whole genome shotgun sequence
Length=7782

 Score = 41.0 bits (44),  Expect = 0.37
 Identities = 25/27 (93%), Gaps = 0/27 (0%)
 Strand=Plus/Minus

Query  1729  CTTGAATATCATATATAAAATGCTTAT  1755
              |||||||||||||||||| ||||| ||
Sbjct  836   CTTGAATATCATATATAATATGCTCAT  810


>emb|CAIC01007804.1| Musa acuminata subsp. malaccensis WGS project CAIC00000000
data,
strain Doubled-haploid Pahang (DH-Pahang), contig_16622,
whole genome shotgun sequence
Length=2817

 Score = 41.0 bits (44),  Expect = 0.37
 Identities = 25/27 (93%), Gaps = 0/27 (0%)
 Strand=Plus/Minus

Query  1729  CTTGAATATCATATATAAAATGCTTAT  1755
              |||||||||||||||| | ||||||||
Sbjct  1869  CTTGAATATCATATATGATATGCTTAT  1843


>emb|CAIC01005535.1| Musa acuminata subsp. malaccensis WGS project CAIC00000000
data,
strain Doubled-haploid Pahang (DH-Pahang), contig_18891,
whole genome shotgun sequence
Length=6544

 Score = 41.0 bits (44),  Expect = 0.37
 Identities = 25/27 (93%), Gaps = 0/27 (0%)
 Strand=Plus/Minus

Query  1729  CTTGAATATCATATATAAAATGCTTAT  1755
              |||||||||||||||||| ||||| ||
```

```
Sbjct  418   CTTGAATATCATATATAATATGCTCAT  392


>emb|CAIC01017495.1| Musa acuminata subsp. malaccensis WGS project CAIC00000000
data,
strain Doubled-haploid Pahang (DH-Pahang), contig_6931,
whole genome shotgun sequence
Length=2901

 Score = 39.2 bits (42),  Expect = 1.3
 Identities = 32/39 (82%), Gaps = 0/39 (0%)
 Strand=Plus/Minus

Query  1938  TCTCTTGGTGATGCATGTCCTTTTATTTTACTAAATGAT  1976
             ||||||||||||||||| | ||| || | | || |||||
Sbjct  439   TCTCTTGGTGATGCATGGCATTTGATATCATTACATGAT  401


>emb|CAIC01016426.1| Musa acuminata subsp. malaccensis WGS project CAIC00000000
data,
strain Doubled-haploid Pahang (DH-Pahang), contig_8000,
whole genome shotgun sequence
Length=28953

 Score = 39.2 bits (42),  Expect = 1.3
 Identities = 23/24 (96%), Gaps = 0/24 (0%)
 Strand=Plus/Plus

Query  1729   CTTGAATATCATATATAAAATGCT  1752
              |||||||||||||||||| |||||
Sbjct  28199  CTTGAATATCATATATAATATGCT  28222


>emb|CAIC01012475.1| Musa acuminata subsp. malaccensis WGS project CAIC00000000
data,
strain Doubled-haploid Pahang (DH-Pahang), contig_11951,
whole genome shotgun sequence
Length=52498

 Score = 39.2 bits (42),  Expect = 1.3
 Identities = 30/35 (86%), Gaps = 2/35 (6%)
 Strand=Plus/Minus

Query  466    TTATGATCAAATTTGTTTATCTATCGAAATTGAAG  500
              ||||| ||||| ||  | |||||||||||||||||
Sbjct  30876  TTATGCTCAAACTT--TAATCTATCGAAATTGAAG  30844


>emb|CAIC01024381.1| Musa acuminata subsp. malaccensis WGS project CAIC00000000
data,
strain Doubled-haploid Pahang (DH-Pahang), contig_44, whole
genome shotgun sequence
Length=341666

 Score = 37.4 bits (40),  Expect = 4.6
```

```
 Identities = 26/30 (87%), Gaps = 0/30 (0%)
 Strand=Plus/Plus

Query  67     CTCCAATCTTCTAATCTTTTCGTATCTAGC  96
              ||||  ||||||||||||| || |||||||
Sbjct  20767  CTCCTCTCTTCTAATCTTTCCGAATCTAGC  20796
```

```
>emb|CAIC01024256.1| Musa acuminata subsp. malaccensis WGS project CAIC00000000
data,
strain Doubled-haploid Pahang (DH-Pahang), contig_169, whole
genome shotgun sequence
Length=87778

 Score = 37.4 bits (40),  Expect = 4.6
 Identities = 37/47 (79%), Gaps = 1/47 (2%)
 Strand=Plus/Minus

Query  1717   AATCATTAGTTACTTGAATATCATATATAAAATGCTTATCTATATCT  1763
              ||| ||||| |  || | |||||||||||||||  ||||  ||||||
Sbjct  23814  AATAATTAGATTTTTAATTATCATATATAAAAT-TTTATTAATATCT  23769
```

```
>emb|CAIC01023384.1| Musa acuminata subsp. malaccensis WGS project CAIC00000000
data,
strain Doubled-haploid Pahang (DH-Pahang), contig_1041,
whole genome shotgun sequence
Length=189108

 Score = 37.4 bits (40),  Expect = 4.6
 Identities = 33/39 (85%), Gaps = 2/39 (5%)
 Strand=Plus/Minus

Query  1255  AGATTCAG-AACCATATGATTCCTGTAGCTACAAAACTC  1292
             |||||||| ||||||||||||||| | |  |||||| ||
Sbjct  8249  AGATTCAGCAACCATATGATTCCT-TCGAAACAAAATTC  8212
```

```
>emb|CAIC01023101.1| Musa acuminata subsp. malaccensis WGS project CAIC00000000
data,
strain Doubled-haploid Pahang (DH-Pahang), contig_1324,
whole genome shotgun sequence
Length=135976

 Score = 37.4 bits (40),  Expect = 4.6
 Identities = 22/23 (96%), Gaps = 0/23 (0%)
 Strand=Plus/Minus

Query  603     GAAGTTATTCTTTTGTAACATCT  625
               |||||||||||||||||||| ||
Sbjct  126381  GAAGTTATTCTTTTGTAACACCT  126359
```

```
>emb|CAIC01022935.1| Musa acuminata subsp. malaccensis WGS project CAIC00000000
data,
```

```
strain Doubled-haploid Pahang (DH-Pahang), contig_1490,
whole genome shotgun sequence
Length=267646

 Score = 37.4 bits (40),  Expect = 4.6
 Identities = 23/25 (92%), Gaps = 0/25 (0%)
 Strand=Plus/Plus

Query  584   TTTCATCTCTTTTGTTTGTGAAGTT  608
             ||||||||  |||||||||||||||
Sbjct  3842  TTTCATCTTCTTTGTTTGTGAAGTT  3866


>emb|CAIC01022901.1| Musa acuminata subsp. malaccensis WGS project CAIC00000000
data,
strain Doubled-haploid Pahang (DH-Pahang), contig_1524,
whole genome shotgun sequence
Length=200714

 Score = 37.4 bits (40),  Expect = 4.6
 Identities = 31/37 (84%), Gaps = 2/37 (5%)
 Strand=Plus/Minus

Query  1014    TTACCAGCCATAATAAGTTTCACAGCTTAACAATATT  1050
               || ||| | ||||||  ||||||||||||| ||||||
Sbjct  156281  TTTCCATCGATAATA--TTTCACAGCTTAATAATATT  156247


>emb|CAIC01022550.1| Musa acuminata subsp. malaccensis WGS project CAIC00000000
data,
strain Doubled-haploid Pahang (DH-Pahang), contig_1875,
whole genome shotgun sequence
Length=202976

 Score = 37.4 bits (40),  Expect = 4.6
 Identities = 25/28 (89%), Gaps = 0/28 (0%)
 Strand=Plus/Minus

Query  1938   TCTCTTGGTGATGCATGTCCTTTTATTT  1965
              |||||| |||||||||||||| || |||
Sbjct  57851  TCTCTTTGTGATGCATGTCCTATTTTTT  57824


 Score = 37.4 bits (40),  Expect = 4.6
 Identities = 28/32 (88%), Gaps = 1/32 (3%)
 Strand=Plus/Minus

Query  1910   TAGGTTGTAT-AAGGTTATTCTCTCCTTGTCT  1940
              |||||||||| || ||  ||||||||||||||
Sbjct  93812  TAGGTTGTATCAATGTGCTTCTCTCCTTGTCT  93781


>emb|CAIC01022177.1| Musa acuminata subsp. malaccensis WGS project CAIC00000000
data,
strain Doubled-haploid Pahang (DH-Pahang), contig_2248,
```

```
whole genome shotgun sequence
Length=58338

 Score = 37.4 bits (40),  Expect = 4.6
 Identities = 22/23 (96%), Gaps = 0/23 (0%)
 Strand=Plus/Minus

Query  595    TTGTTTGTGAAGTTATTCTTTTG  617
              |||||||| ||||||||||||||
Sbjct  38464  TTGTTTGTTAAGTTATTCTTTTG  38442


>emb|CAIC01021950.1| Musa acuminata subsp. malaccensis WGS project CAIC00000000
data,
strain Doubled-haploid Pahang (DH-Pahang), contig_2475,
whole genome shotgun sequence
Length=153504

 Score = 37.4 bits (40),  Expect = 4.6
 Identities = 25/28 (89%), Gaps = 0/28 (0%)
 Strand=Plus/Plus

Query  593    TTTTGTTTGTGAAGTTATTCTTTTGTAA  620
              |||| ||||||||||| ||||||| |||
Sbjct  15915  TTTTTTTTGTGAAGTTTTTCTTTTATAA  15942


>emb|CAIC01021109.1| Musa acuminata subsp. malaccensis WGS project CAIC00000000
data,
strain Doubled-haploid Pahang (DH-Pahang), contig_3317,
whole genome shotgun sequence
Length=183738

 Score = 37.4 bits (40),  Expect = 4.6
 Identities = 31/37 (84%), Gaps = 2/37 (5%)
 Strand=Plus/Plus

Query  1731    TGAATATCATATATAAAATGCTTATCTATATCTGTTT  1767
               || |||| |||||||||||||  || ||||||| |||
Sbjct  157642  TGTATATAATATATAAAATGC--ATATATATCTTTTT  157676


>emb|CAIC01020790.1| Musa acuminata subsp. malaccensis WGS project CAIC00000000
data,
strain Doubled-haploid Pahang (DH-Pahang), contig_3636,
whole genome shotgun sequence
Length=26865

 Score = 37.4 bits (40),  Expect = 4.6
 Identities = 20/20 (100%), Gaps = 0/20 (0%)
 Strand=Plus/Minus

Query  1948  ATGCATGTCCTTTTATTTTA  1967
             ||||||||||||||||||||
Sbjct  6857  ATGCATGTCCTTTTATTTTA  6838
```

```
>emb|CAIC01019226.1| Musa acuminata subsp. malaccensis WGS project CAIC00000000
data,
strain Doubled-haploid Pahang (DH-Pahang), contig_5200,
whole genome shotgun sequence
Length=91398

 Score = 37.4 bits (40),  Expect = 4.6
 Identities = 23/25 (92%), Gaps = 0/25 (0%)
 Strand=Plus/Minus

Query  1240  AGGTTATGAGCTCATAGATTCAGAA  1264
             || |||||| |||||||||||||||
Sbjct  4894  AGATTATGAACTCATAGATTCAGAA  4870


>emb|CAIC01018551.1| Musa acuminata subsp. malaccensis WGS project CAIC00000000
data,
strain Doubled-haploid Pahang (DH-Pahang), contig_5875,
whole genome shotgun sequence
Length=6935

 Score = 37.4 bits (40),  Expect = 4.6
 Identities = 25/28 (89%), Gaps = 0/28 (0%)
 Strand=Plus/Minus

Query  1132  CATCAATGCAGTGTACATAGTGAGTGAT  1159
             |||||||| ||| ||| |||||||||||
Sbjct  174   CATCAATGTAGTTTACGTAGTGAGTGAT  147


>emb|CAIC01018434.1| Musa acuminata subsp. malaccensis WGS project CAIC00000000
data,
strain Doubled-haploid Pahang (DH-Pahang), contig_5992,
whole genome shotgun sequence
Length=47185

 Score = 37.4 bits (40),  Expect = 4.6
 Identities = 25/28 (89%), Gaps = 0/28 (0%)
 Strand=Plus/Plus

Query  554    TTTTATATTTGAAGTCttttttttCCCT  581
              |||||||||||| |||||| ||||| ||
Sbjct  19143  TTTTATATTTGATGTCTTTATTTTCACT  19170


>emb|CAIC01015797.1| Musa acuminata subsp. malaccensis WGS project CAIC00000000
data,
strain Doubled-haploid Pahang (DH-Pahang), contig_8629,
whole genome shotgun sequence
Length=81298

 Score = 37.4 bits (40),  Expect = 4.6
 Identities = 25/27 (93%), Gaps = 1/27 (4%)
```

```
 Strand=Plus/Plus

Query  1979   GACCAAAACCTTCTTTTGAAACATAAC  2005
              ||||||| |||||||||||||| ||||
Sbjct  24129  GACCAAATCCTTCTTTTGAAAC-TAAC  24154


>emb|CAIC01014197.1| Musa acuminata subsp. malaccensis WGS project CAIC00000000
data,
strain Doubled-haploid Pahang (DH-Pahang), contig_10229,
whole genome shotgun sequence
Length=44984

 Score = 37.4 bits (40),  Expect = 4.6
 Identities = 26/30 (87%), Gaps = 0/30 (0%)
 Strand=Plus/Minus

Query  1989  TTCTTTTGAAACATAACTGGAAACCCGAAT  2018
             ||||||||||| |||||||||   ||||||
Sbjct  8446  TTCTTTTGAAAGATAACTGGAGCTCCGAAT  8417


>emb|CAIC01013428.1| Musa acuminata subsp. malaccensis WGS project CAIC00000000
data,
strain Doubled-haploid Pahang (DH-Pahang), contig_10998,
whole genome shotgun sequence
Length=35152

 Score = 37.4 bits (40),  Expect = 4.6
 Identities = 26/30 (87%), Gaps = 0/30 (0%)
 Strand=Plus/Plus

Query  1727  TACTTGAATATCATATATAAAATGCTTATC  1756
             ||||| ||| || ||| |||||||||||||
Sbjct  4860  TACTTAAATTTCTTATTTAAAATGCTTATC  4889


>emb|CAIC01012759.1| Musa acuminata subsp. malaccensis WGS project CAIC00000000
data,
strain Doubled-haploid Pahang (DH-Pahang), contig_11667,
whole genome shotgun sequence
Length=28673

 Score = 37.4 bits (40),  Expect = 4.6
 Identities = 31/37 (84%), Gaps = 2/37 (5%)
 Strand=Plus/Plus

Query  461   TATCGTTATGATCAAATTTGTTTATCTATCGAAATTG  497
             |||||||||||||||  || ||  |||||| ||||||
Sbjct  6305  TATCGTTATGATCAA--TTTTTAGTCTATCAAAATTG  6339


>emb|CAIC01011690.1| Musa acuminata subsp. malaccensis WGS project CAIC00000000
data,
strain Doubled-haploid Pahang (DH-Pahang), contig_12736,
```

```
whole genome shotgun sequence
Length=26610

 Score = 37.4 bits (40),  Expect = 4.6
 Identities = 23/25 (92%), Gaps = 0/25 (0%)
 Strand=Plus/Plus

Query  1727   TACTTGAATATCATATATAAAATGC  1751
              |||||||||||||||||| | ||||
Sbjct  21068  TACTTGAATATCATATATGATATGC  21092


>emb|CAIC01010724.1| Musa acuminata subsp. malaccensis WGS project CAIC00000000
data,
strain Doubled-haploid Pahang (DH-Pahang), contig_13702,
whole genome shotgun sequence
Length=11238

 Score = 37.4 bits (40),  Expect = 4.6
 Identities = 31/38 (82%), Gaps = 0/38 (0%)
 Strand=Plus/Plus

Query  1718  ATCATTAGTTACTTGAATATCATATATAAAATGCTTAT  1755
             || |||| | ||||||||||||| || || ||||| ||
Sbjct  3112  ATAATTAATGACTTGAATATCATGTACAATATGCTCAT  3149


>emb|CAIC01009744.1| Musa acuminata subsp. malaccensis WGS project CAIC00000000
data,
strain Doubled-haploid Pahang (DH-Pahang), contig_14682,
whole genome shotgun sequence
Length=23865

 Score = 37.4 bits (40),  Expect = 4.6
 Identities = 26/30 (87%), Gaps = 0/30 (0%)
 Strand=Plus/Minus

Query  629   GCAAATCCTACAACTTCACCAGCTACTGGT  658
             ||||||| ||||||| |  |||||||||||
Sbjct  7504  GCAAATCTTACAACTACTTCAGCTACTGGT  7475


>emb|CAIC01008714.1| Musa acuminata subsp. malaccensis WGS project CAIC00000000
data,
strain Doubled-haploid Pahang (DH-Pahang), contig_15712,
whole genome shotgun sequence
Length=39168

 Score = 37.4 bits (40),  Expect = 4.6
 Identities = 29/34 (85%), Gaps = 2/34 (6%)
 Strand=Plus/Plus

Query  1023   ATAATAAGTTTCACAGCTTAACAATATTCATATA  1056
              || ||||| |||||  ||||| ||||||||||||
Sbjct  19925  ATTATAAGATTCAC--CTTAAGAATATTCATATA  19956
```

```
  Database: WGS_VDB://CAIC01
    Posted date:
  Number of letters in database: 390,578,572
  Number of sequences in database:  24,424

Lambda      K        H
   0.634    0.408    0.912
Gapped
Lambda      K        H
   0.625    0.410    0.780
Matrix: blastn matrix:2 -3
Gap Penalties: Existence: 5, Extension: 2
Number of Sequences: 24424
Number of Hits to DB: 10076462
Number of extensions: 19883
Number of successful extensions: 19883
Number of sequences better than 10: 46
Number of HSP's better than 10 without gapping: 0
Number of HSP's gapped: 19883
Number of HSP's successfully gapped: 47
Length of query: 2087
Length of database: 390578572
Length adjustment: 31
Effective length of query: 2056
Effective length of database: 389821428
Effective search space: 801472855968
Effective search space used: 801472855968
A: 0
X1: 22 (20.1 bits)
X2: 33 (29.8 bits)
X3: 110 (99.2 bits)
S1: 28 (26.5 bits)
S2: 39 (36.5 bits)
```
