# Supplementary material for: Expressed Centromere Specific Histone 3 (CENH3) Variants in Cultivated Triploid and Wild Diploid Bananas (Musa spp.)
Source: Front Plant Sci. 2017 Jun 29;8:1034. doi: 10.3389/fpls.2017.01034 (PMC5489561; doi:10.3389/fpls.2017.01034)
Supplement: Supplementary file 2 [file Data_Sheet_2.pdf]

```
                        1         10        20        30        40        50        60
                        |         |         |         |         |         |         |
    M. balbisiana-10   ATGGCGAGAACGAAGCATCTGTCCAACAGGTCCTCCTCTCGCCCTCGGAAGCGCTTCCAT
       Gros Michel-2   ATGGCGAGAACGAAGCATCTGTCCAACAGGTCCTCCTCTCGCCCTCGGAAGCGCTTCCAT
       Pisang Awak-4   ATGGCGAGAACGAAGCATCTGTCCAACAGGTCCTCCTCTCGCCCTCGGAAGCGCTTCCAT
     Sukali Ndiizi-A   ATGGCGAGAACGAAGCATCTGTCCAACAGGTCCTCCTCTCGCCCTCGGAAGCGCTTCCAT
        Zebrina GF-8   ATGGCGAGAACGAAGCATCTGTCCAACAGGTCCCCCTCTCGCCCTCGGAAGCGCTTCCAT
       Calcutta 4-11   ATGGCGAGAACGAAGCATCTGTCCAACAGGTCCTCCTCTCGCCCTCGGAAGCGCTTCCAT
       Gros_Michel-1   ATGGCGAGAACGAAGCATCTGTCCAACAGGTCCTCCTCTCGCCCTCGGAAGCGCTTCCAT
     Sukali Ndiizi-H   ATGGCGAGAACGAAGCATCTGTCCAACAGGTCCTCCTCTCGCCCTCGGAAGCGCTTCCAT
        Zebrina GF-6   ATGGCGAGAACGAAGCATCTGTCCAATAGGTCCTCCTCTCGCCCTCGGAAGCGCTTCCAT
        Zebrina GF-7   ATGGCGAGAACGAAGCATCTGTCCAATAGGTCCTCCTCTCGCCCTCGGAAGCGCTTCCAT
       Pisang Awak-5   ATGGCGAGAACGAAGCATCTGTCCAACAGGTCCTCCTCTCGCCCTCGGAAGCGCTTCCAT
     Sukali Ndiizi-F   ATGGCGAGAACGAAGCATCTGTCCAACAGGTCCTCCTCTCGCCCTCGGAAGCGCTTCCAT
     Sukali Ndiizi-G   ATGGCGAGAACGAAGCATCTGTCCAACAGGTCCTCCTCTCGCCCTCGGAAGCGCTTCCAT
        Zebrina GF-9   ATGGCGAGAACGAAGCATCTGTCCAACAGGTCCTCCTCTCGCCCTCGGAAGCGCTTCCAT
     Sukali Ndiizi-E   ATGGCGAGAACGAAGCATCTGTCCAACAGGTCCTCCTCTCGCCCTCGGAAGCGCTTCCAT
     Sukali Ndiizi-C   ATGGCGAGAACGAAGCATCTGTCCAACAGGTCCTCCTCTCGCCCTCGGAAGCGCTTCCAT
     Sukali Ndiizi-B   ATGGCGAGAACGAAGCATCTGTCCAACAGGTCCTCCTCTCGCCCTCGGAAGCGCTTCCAT
M. acuminata genomic   ATGGCGAGAACGAAGCATCTGTCCAACAGGTCCTCCTCTCGCCCTCGGAAGCGCTTCCAT

    M. balbisiana-10   TTCGGTCGGTCTCCAGGGCAGCGAACCCCCG-----------------------------
       Gros Michel-2   TTCGGTCGGTCTCCAGGGCAGCGAACCCCCG-----------------------------
       Pisang Awak-4   TTCGGTCGGTCTCCAGGGCAGCGAACCCCCG-----------------------------
     Sukali Ndiizi-A   TTCGGTCGGTCTCCAGGGCAGCGAACCCCCG-----------------------------
        Zebrina GF-8   TTCGGTCGGTCTCCAGGGCAGCGAACCCCCG-----------------------------
       Calcutta 4-11   TTCGGTCGGTCTCCAGGGCAGCGAACCCCCG-----------------------------
       Gros_Michel-1   TTCGGTCGGTCTCCAGGGCAGCGAACCCCCG-----------------------------
     Sukali Ndiizi-H   TTCGGTCGGTCTCCAGGGCAGCGAACCCCCG-----------------------------
        Zebrina GF-6   TTCGGTCGGTCTCCAGGGCAGCGAACCCCCG-----------------------------
        Zebrina GF-7   TTCGGTCGGTCTCCAGGGCAGCGAACCCCCG-----------------------------
       Pisang Awak-5   TTCGGTCGGTCTCCAGGGCAGCGAACCCCCG-----------------------------
     Sukali Ndiizi-F   TT---------------------------CG-----------------------------
     Sukali Ndiizi-G   TTCGGTCGGTCTCCAGGGCAGCGAACCCCCG-----------------------------
        Zebrina GF-9   TTCGGTCGGTCTCCAGGGCGGCGAACCCCCG-----------------------------
     Sukali Ndiizi-E   TTCGGTCGGTCTCCAGGGCAGCGAACCCCCG-----------------------------
     Sukali Ndiizi-C   TTCGGTCGGTCTCCAGGGCAGCGAACCCCCG-----------------------------
     Sukali Ndiizi-B   TTCGGTCGGTCTCCAGGGCAGCGAACCCCCG-----------------------------
M. acuminata genomic   TTCGGTCGGTCTCCAGGGCAGCGAACCCCCGGTCCGCCCATCTCTCCTTTTCTCCTCTCT

    M. balbisiana-10   ------------------------------------------------------------
       Gros Michel-2   ------------------------------------------------------------
       Pisang Awak-4   ------------------------------------------------------------
     Sukali Ndiizi-A   ------------------------------------------------------------
        Zebrina GF-8   ------------------------------------------------------------
       Calcutta 4-11   ------------------------------------------------------------
       Gros_Michel-1   ------------------------------------------------------------
     Sukali Ndiizi-H   ------------------------------------------------------------
        Zebrina GF-6   ------------------------------------------------------------
        Zebrina GF-7   ------------------------------------------------------------
       Pisang Awak-5   ------------------------------------------------------------
     Sukali Ndiizi-F   ------------------------------------------------------------
     Sukali Ndiizi-G   ------------------------------------------------------------
        Zebrina GF-9   ------------------------------------------------------------
     Sukali Ndiizi-E   ------------------------------------------------------------
     Sukali Ndiizi-C   ------------------------------------------------------------
     Sukali Ndiizi-B   ------------------------------------------------------------
M. acuminata genomic   TCTTTGGTTTGTTGGTGTTAGATTTCTTCTGATCTCTTTCCTTGCCTCTTTTCATTGTTA

    M. balbisiana-10   ----CTGATGCGAATCGGCCTGCGACGCCATCTGGTGCTACTCCT---------------
       Gros Michel-2   ----CTGATGCGAATCGGCCTGCGACGCCATCTGGTGCTACTCCT---------------
```

```
       Pisang Awak-4   ----CTGATGCGAATCGGCCTGCGACGCCATCTGGTGCTACTCCT---------------
     Sukali Ndiizi-A   ----CTGATGCGAATCGACCTGCGACGCCCTCTGGTGCTACTCCT---------------
        Zebrina GF-8   ----CTGATGCGAATCGACCTGCGACGCCATCTGGTGCTACTCCT---------------
       Calcutta 4-11   ----CTGATGCGAATCGACCTGCGACGCCATCTGGTGCTACTCCT---------------
       Gros_Michel-1   ----CTGATGCGAATCGACCTGCGACGCCATCTGGTGCTACTCCT---------------
     Sukali Ndiizi-H   ----CTGATGCGAATCGACCTGCGACGCCATCTGGTGCTACTCCT---------------
        Zebrina GF-6   ----CTGATGCGAATCGACCTGCGACGCCATCTGGTGCTACTCCT---------------
        Zebrina GF-7   ----CTGATGCGAATCGACCTGCGACGCCATCTGGTGCTACTCCT---------------
       Pisang Awak-5   ----CTGATGCGAATCGACCTGCGACGCCATCTGGTGCTACTCCTGTGCGCTTCTCTTCT
     Sukali Ndiizi-F   ----CTGATGCGAATCGACCTGCGACGCCATCTGGTGCTACTCCT---------------
     Sukali Ndiizi-G   ----CTGATGCGAATCGACCTGCGACGCCATCTGGTGCTACTCCT---------------
        Zebrina GF-9   -------------GTCCGCCCATCTCTCCTTTTCTCCTCTCTTCT---------------
     Sukali Ndiizi-E   -------------GTCCGCCCATCTCTCCTTTTCTCCTCTCTTCT---------------
     Sukali Ndiizi-C   -------------GTCCGCCCATCTCTCCTTTTCTCCTCTCTTCT---------------
     Sukali Ndiizi-B   -------------GTCCGCCCATCTCTCCTTTTCTCCTCTCTTCT---------------
M. acuminata genomic   ATAGCTGATGCGAATCGACCTGCGACGCCATCTGGTGCTACTCCTGTGCGCTTCTCTTCT

    M. balbisiana-10   ------------------------------------------------------------
       Gros Michel-2   ------------------------------------------------------------
       Pisang Awak-4   ------------------------------------------------------------
     Sukali Ndiizi-A   ------------------------------------------------------------
        Zebrina GF-8   ------------------------------------------------------------
       Calcutta 4-11   ------------------------------------------------------------
       Gros_Michel-1   ------------------------------------------------------------
     Sukali Ndiizi-H   ------------------------------------------------------------
        Zebrina GF-6   ------------------------------------------------------------
        Zebrina GF-7   ------------------------------------------------------------
       Pisang Awak-5   CACTATTCTTTCCTTCTTCCTCGTTTCTTTGGATTTTTAAGCTTGATTGTCTTGTATATT
     Sukali Ndiizi-F   ------------------------------------------------------------
     Sukali Ndiizi-G   ------------------------------------------------------------
        Zebrina GF-9   ------------------------------------------------------------
     Sukali Ndiizi-E   ------------------------------------------------------------
     Sukali Ndiizi-C   ------------------------------------------------------------
     Sukali Ndiizi-B   ------------------------------------------------------------
M. acuminata genomic   CACTATTCTTTCCTTCTTCCTCGTTTCTTTGGATTTTTAAGCTTGATTGTCTTGTATATT

    M. balbisiana-10   ------------AGAACCACGGCCACCAGATCGAGGGATACGCCTCA-------------
       Gros Michel-2   ------------AGAACCACGGCCACCAGATCGAGGGATACGCCTCA-------------
       Pisang Awak-4   ------------AGAACCACGGCCACCAGATCGAGGGATACGCCTCA-------------
     Sukali Ndiizi-A   ------------AGAACCACGGCCACCAGATCGAGGAACGCGCCTCA-------------
        Zebrina GF-8   ------------AGAACCACGGCCACCAGATCGAGGAACGCGCCTCA-------------
       Calcutta 4-11   ------------AGAACCACGGCCACCAGATCGAGGAACGCGCCTCA-------------
       Gros_Michel-1   ------------AGAACCACGGCCACCAGATCGAGGAACGCGCCTCA-------------
     Sukali Ndiizi-H   ------------AGAACCACGGCCACCAGATCGAGGAACGCGCCTCA-------------
        Zebrina GF-6   ------------AGAACCACGGCCACCAGATCGAGGAACGCGCCTCA-------------
        Zebrina GF-7   ------------AGAACCACGGCCACCAGATCGAGGAACGCGCCTCA-------------
       Pisang Awak-5   TCGCACGAATAGAGAACCACGGCCACCAGATCGAGGAACGCGCCTCAAGGTCTTCACCTT
     Sukali Ndiizi-F   ------------AGAACCACGGCCACCAGATCGAGGAACGCGCCTCA-------------
     Sukali Ndiizi-G   ------------GC----------------------------------------------
        Zebrina GF-9   -------------------------TTGGTTTG-----------TTG-------------
     Sukali Ndiizi-E   -------------------------TTGGTTTG-----------TTG-------------
     Sukali Ndiizi-C   -------------------------TTGGTTTG-----------TTG-------------
     Sukali Ndiizi-B   -------------------------TTGGTTTG-----------TTG-------------
M. acuminata genomic   TCGCACGAATAGAGAACCACGGCCACCAGATCGAGGAACGCGCCTCAAGGTCTTCACCTT

    M. balbisiana-10   ------------------------------------------------------------
       Gros Michel-2   ------------------------------------------------------------
       Pisang Awak-4   ------------------------------------------------------------
     Sukali Ndiizi-A   ------------------------------------------------------------
        Zebrina GF-8   ------------------------------------------------------------
       Calcutta 4-11   ------------------------------------------------------------
       Gros_Michel-1   ------------------------------------------------------------
     Sukali Ndiizi-H   ------------------------------------------------------------
        Zebrina GF-6   ------------------------------------------------------------
```

```
        Zebrina GF-7   ------------------------------------------------------------
       Pisang Awak-5   TTCGTACTTCTTGCGCTTGATTATATGTGATTATTTATTTATTTTTTCTTACGCGCACAC
     Sukali Ndiizi-F   ------------------------------------------------------------
     Sukali Ndiizi-G   ------------------------------------------------------------
        Zebrina GF-9   ------------------------------------------------------------
     Sukali Ndiizi-E   ------------------------------------------------------------
     Sukali Ndiizi-C   ------------------------------------------------------------
     Sukali Ndiizi-B   ------------------------------------------------------------
M. acuminata genomic   TTCGTACTTCTTGCGCTTGATTATATGTGATTATTTATTTATTTTTTCTTACGCGCACAC

    M. balbisiana-10   ------------------------------------------------------------
       Gros Michel-2   ------------------------------------------------------------
       Pisang Awak-4   ------------------------------------------------------------
     Sukali Ndiizi-A   ------------------------------------------------------------
        Zebrina GF-8   ------------------------------------------------------------
       Calcutta 4-11   ------------------------------------------------------------
       Gros_Michel-1   ------------------------------------------------------------
     Sukali Ndiizi-H   ------------------------------------------------------------
        Zebrina GF-6   ------------------------------------------------------------
        Zebrina GF-7   ------------------------------------------------------------
       Pisang Awak-5   CAAATAAGTTCTTATTAGATAAAAGGGGAAAAAAAAGAAGGAAAAAGAGGTCGGAATTTG
     Sukali Ndiizi-F   ------------------------------------------------------------
     Sukali Ndiizi-G   ------------------------------------------------------------
        Zebrina GF-9   ------------------------------------------------------------
     Sukali Ndiizi-E   ------------------------------------------------------------
     Sukali Ndiizi-C   ------------------------------------------------------------
     Sukali Ndiizi-B   ------------------------------------------------------------
M. acuminata genomic   CAAATAAGTTCTTATTAGATAAAAGGGGAAAAAAAAGAAGGAAAAAGAGGTCGGAATTTG

    M. balbisiana-10   ------------------------------------AGGGGCACCGAGCCAATCAAAGCA
       Gros Michel-2   ------------------------------------AGGGGCACCGAGCCAATCAAAACA
       Pisang Awak-4   ------------------------------------AGGGGCACCGAGCCAATCAAAGCA
     Sukali Ndiizi-A   ------------------------------------AGGGGCACCGAGCCAATCAAAGAA
        Zebrina GF-8   ------------------------------------AGGGGCACCGAGCCAATCAAAGAA
       Calcutta 4-11   ------------------------------------AGGGGCACCGAGCCAATCAAAGAA
       Gros_Michel-1   ------------------------------------AGGGGCACCGAGCCAATCAAAGAA
     Sukali Ndiizi-H   ------------------------------------AGGGGCACCGAGCCAATCAAAGAA
        Zebrina GF-6   ------------------------------------AGGGGCACCGAGCCAATCAAAGAA
        Zebrina GF-7   ------------------------------------AGGGGCACCGAGCCAATCAAAGAA
       Pisang Awak-5   AATGAAAAGGGTTCCCTTTTTTTGTTGGGTGGAGGCAGGGGCACCGAGCCAATCAAAGAA
     Sukali Ndiizi-F   ------------------------------------AGGGGCACCGAGCCAACCAAAGAA
     Sukali Ndiizi-G   ------------------------------------AGGGGCACCGAGCCAATCAAAGAA
        Zebrina GF-9   ------------------------------------GTGGGCACCGAGCCAATCAAAGAA
     Sukali Ndiizi-E   ------------------------------------GTGGGCACCGAGCCAATCAAAGAA
     Sukali Ndiizi-C   ------------------------------------GTGGGCACCGAGCCAATCAAAGAA
     Sukali Ndiizi-B   ------------------------------------GTGGGCACCGAGCCAATCAAAGAA
M. acuminata genomic   AATGAAAAGGGTTCCCTTTTTTTGTTGGGTGGAGGCAGGGGCACCGAGCCAATCAAAGAA

    M. balbisiana-10   GCAGCCGAGGCGGCGCAGGTTTAGGCCGGGGGTGGTGGCGCTACGCGAGATCAGGAATTT
       Gros Michel-2   GCAGCCGAGGCGGCGCAGGTTTAGGCCGGGGGTGGTGGCGCTACGCGAGATCAGGAATTT
       Pisang Awak-4   GCAGCCGAGGCGGCGCAGGTTTAGGCCGGGGGTGGTGGCGCTACGCGAGATCAGGAATTT
     Sukali Ndiizi-A   GCAGCCGAGGCGGCGCAGGTTTAGGCCGGGGGTGGTGGCGCTACGCGAGATCAGGAATTT
        Zebrina GF-8   GCAGCCGAGGCGGCGCAGGTTTAGGCCGGGGGTGGTGGCGCTACGCGAGATCAGGAATTT
       Calcutta 4-11   GCAGCCGAGGCGGCGCAGGTTTAGGCCGGGGGTGGTGGCGCTACGCGAGATCAGGAATTT
       Gros_Michel-1   GCAGCCGAGGCGGCGCAGGTTTAGGCCGGGGGTGGTGGCGCTACGCGAGATCAGGAATTT
     Sukali Ndiizi-H   GCAGCCGAGGCGGCGCAGGTTTAGGCCGGGGGTGGTGGCGCTACGCGAGATCAGGAATTT
        Zebrina GF-6   GCAGCCGAGGCGGCGCAGGTTTAGGCCGGGGGTGGTGGCGCTACGCGAGATCAGGAATTT
        Zebrina GF-7   GCAGCCGAGGCGGCGCAGGTTTAGGCCGGGGGTGGTGGCGCTACGCGAGATCAGGAATTT
       Pisang Awak-5   GCAGCCGAGGCGGCGCAGGTTTAGGCCGGGGGTGGTGGCGCTACGCGAGATCAGGAATTT
     Sukali Ndiizi-F   GCAGCCGAGGCGGCGCAGGTTTAGGCCGGGGGTGGTGGCGCTACGCGAGATCAGGAATTT
     Sukali Ndiizi-G   GCAGCCGAGGCGGCGCAGGTTTAGGCCGGGGGTGGTGGCGCTACGCGAGATCAGGAATTT
        Zebrina GF-9   GCAGCCGAGGCGGCGCAGGTTTAGGCCGGGGGTGGTGGCGCTACGCGAGATCAGGAATTT
     Sukali Ndiizi-E   GCAGCCGAGGCGGCGCAGGTTTAGGCCGGGGGTGGTGGCGCTACGCGAGATCAGGAATTT
     Sukali Ndiizi-C   GCAGCCGAGGCGGCGCAGGTTTAGGCCGGGGGTGGTGGCGCTACGCGGGATCAGGAATTT
```

```
      Sukali Ndiizi-B   GCAGCCGAGGCGGCGCAGGTTTAGGCCGGGGGTGGTGGCGCTACGCGAGATCAGGAATTT
M. acuminata genomic   GCAGCCGAGGCGGCGCAGGTTTAGGCCGGGGGTGGTGGCGCTACGCGAGATCAGGAATTT

     M. balbisiana-10   GCAGAAGACGTGGAATCTATTGATCCCTTTCGCTCCGTTTGTCAGACTTG----------
        Gros Michel-2   GCAGAAGACGTGGAATCTATTGATCCCTTTCGCTCCGTTTGTCAGACTTG----------
        Pisang Awak-4   GCAGAAGACGTGGAATCTATTGATCCCTTTCGCTCCGTTTGTCAGACTTG----------
      Sukali Ndiizi-A   GCAGAAGACGTGGAATCTATTGATCCCTTTCGCTCCGTTTGTCAGACTTG----------
         Zebrina GF-8   GCAGAAGACGTGGAATCTATTGATCCCTTTCGCTCCGTTTGTCAGACTTG----------
        Calcutta 4-11   GCAGAAGACGTGGAATCTATTGATCCCTTTCGCTCCGTTTGTCAGACTTG----------
        Gros_Michel-1   GCAGAAGACGTGGAATCTATTGATCCCTTTCGCTCCGTTTGTCAGACTTG----------
      Sukali Ndiizi-H   GCAGAAGACGTGGAATCTATTGATCCCTTTCGCTCCGTTTGTCAGACTTG----------
         Zebrina GF-6   GCAGAAGACGTGGAATCTATTGATCCCTTTCGCTCCGTTTGTCAGACTTG----------
         Zebrina GF-7   GCAGAAGACGTGGAATCTATTGATCCCTTTCGCTCCGTTTGTCAGACTTG----------
        Pisang Awak-5   GCAGAAGACGTGGAATCTATTGATCCCTTTCGCTCCGTTTGTCAGACTTG----------
      Sukali Ndiizi-F   GCAGAAGACGTGGAATCTATTGATCCCTTTCGCTCCGTTTGTCAGACTTG----------
      Sukali Ndiizi-G   GCAGAAGACGTGGAATCTATTGATCCCTTTCGCTCCGTTTGTCAGACTTG----------
         Zebrina GF-9   GCAGAAGACGTGGAATCTATTGATCCCTTTCGCTCCGTTTGTCAGACTTG----------
      Sukali Ndiizi-E   GCAGAAAACGTGGAATCTATTGATCCCTTTCGCTCCGTTTGTCAGACTTG----------
      Sukali Ndiizi-C   GCAGAAGACGTGGAATCTATTGATCCCTTTCGCTCCGTTTGTCAGACTTG----------
      Sukali Ndiizi-B   GCAGAAGACGTGGAATCTATTGATCCCTTTCGCTCCGTTTGTCAGACTTG----------
M. acuminata genomic   GCAGAAGACGTGGAATCTATTGATCCCTTTCGCTCCGTTTGTCAGACTTGTTAGTACCAT

     M. balbisiana-10   ------------------------------------------------------------
        Gros Michel-2   ------------------------------------------------------------
        Pisang Awak-4   ------------------------------------------------------------
      Sukali Ndiizi-A   ------------------------------------------------------------
         Zebrina GF-8   ------------------------------------------------------------
        Calcutta 4-11   ------------------------------------------------------------
        Gros_Michel-1   ------------------------------------------------------------
      Sukali Ndiizi-H   ------------------------------------------------------------
         Zebrina GF-6   ------------------------------------------------------------
         Zebrina GF-7   ------------------------------------------------------------
        Pisang Awak-5   ------------------------------------------------------------
      Sukali Ndiizi-F   ------------------------------------------------------------
      Sukali Ndiizi-G   ------------------------------------------------------------
         Zebrina GF-9   ------------------------------------------------------------
      Sukali Ndiizi-E   ------------------------------------------------------------
      Sukali Ndiizi-C   ------------------------------------------------------------
      Sukali Ndiizi-B   ------------------------------------------------------------
M. acuminata genomic   CATCGTCTGTATTTTCCCCTTTTTGCAACAATAATGCAGAAAAAAATACGTTCTCCAAGC

     M. balbisiana-10   ------------------------------------------------------------
        Gros Michel-2   ------------------------------------------------------------
        Pisang Awak-4   ------------------------------------------------------------
      Sukali Ndiizi-A   ------------------------------------------------------------
         Zebrina GF-8   ------------------------------------------------------------
        Calcutta 4-11   ------------------------------------------------------------
        Gros_Michel-1   ------------------------------------------------------------
      Sukali Ndiizi-H   ------------------------------------------------------------
         Zebrina GF-6   ------------------------------------------------------------
         Zebrina GF-7   ------------------------------------------------------------
        Pisang Awak-5   ------------------------------------------------------------
      Sukali Ndiizi-F   ------------------------------------------------------------
      Sukali Ndiizi-G   ------------------------------------------------------------
         Zebrina GF-9   ------------------------------------------------------------
      Sukali Ndiizi-E   ------------------------------------------------------------
      Sukali Ndiizi-C   ------------------------------------------------------------
      Sukali Ndiizi-B   ------------------------------------------------------------
M. acuminata genomic   TCCGTAGATGCTGCTCTGGGGATGACTTGTGGTTTTGGGTTTAGAAAGGTTTACATGTTC

     M. balbisiana-10   --------------------------------TTCGGGAGATCACTCATTTCTACTCGAA
        Gros Michel-2   --------------------------------TTCGGGAGATCACTCATTTCTACTCGAA
        Pisang Awak-4   --------------------------------TACGGGAGATCACTCATTTCTACTCGAA
      Sukali Ndiizi-A   --------------------------------TACGGGAGATCACTCATTTCTACTCGAA
```

```
         Zebrina GF-8   --------------------------------TACGGGAGATCACTCATTTCTACTCGAA
        Calcutta 4-11   --------------------------------TACGGGAGATCACTCATTTCTACTCGAA
        Gros_Michel-1   --------------------------------TACGGGAGATCACTCATTTCTACTCGAA
      Sukali Ndiizi-H   --------------------------------TACGGGAGATCACTCATTTCTACTCGAA
         Zebrina GF-6   --------------------------------TACGGGAGATCACTCATTTCTACTCGAA
         Zebrina GF-7   --------------------------------TACGGGAGATCACTCATTTCTACTCGAA
        Pisang Awak-5   --------------------------------TACGGGAGATCACTCATTTCTACTCGAA
      Sukali Ndiizi-F   --------------------------------TACGGGAGATCACTCATTTCTACTCGAA
      Sukali Ndiizi-G   --------------------------------TACGGGAGATCACTCATTTCTACTCGAA
         Zebrina GF-9   --------------------------------TACGGGAGATCACTCATTTCTACTCGAA
      Sukali Ndiizi-E   --------------------------------TACGGGAGATCACTCATTTCTACTCGAA
      Sukali Ndiizi-C   --------------------------------TACGGGGGATCACTCATTTCTACTCGAA
      Sukali Ndiizi-B   --------------------------------TACGGGAGATCACTCATTTCTACTCGAA
M. acuminata genomic    TACTTCTAATGATGTTTGTCAGTGGATCTAGGTACGGGAGATCACTCATTTCTACTCGAA

     M. balbisiana-10   AGAAGTAAACCGATGGACCCCTGAAGCTTTGGTTGCGATTCAAG----------------
        Gros Michel-2   AGAAGTAAACCGATGGACCCCTGAAGCTTTAGTTGCGATTCAAG----------------
        Pisang Awak-4   AGAAGTAAACCGATGGACCCCTGAAGCTTTGGTTGCAATTCAAG----------------
      Sukali Ndiizi-A   AGAAGTAAACCGATGGACCCCTGAAGCTTTGGTTGCGATTCAAG----------------
         Zebrina GF-8   AGAAGTAAACCGATGGACCCCTGAAGCTTTGGTTGCGATTCAAG----------------
        Calcutta 4-11   AGAAGTAAACCGATGGACCCCTGAAGCTTTGGTTGCGATTCAAG----------------
        Gros_Michel-1   AGAAGTAAACCGATGGACCCCTGAAGCTTTGGTTGCGATTCAAG----------------
      Sukali Ndiizi-H   AGAAGTAAACCGATGGACCCCTGAAGCTTTGGTTGCGATTCAAG----------------
         Zebrina GF-6   AGAAGTAAACCGATGGACCCCTGAAGCTTTGGTTGCGATTCAAG----------------
         Zebrina GF-7   AGAAGTAAACCGATGGACCCCTGAAGCTTTGGTTGCGATTCAAG----------------
        Pisang Awak-5   AGAAGTAAACCGATGGACCCCTGAAGCTTTGGTTGCGATTCAAG----------------
      Sukali Ndiizi-F   AGAAGTAAACCGATGGACCCCTGAAGCTTTGGTTGCGATTCAAG----------------
      Sukali Ndiizi-G   AGAAGTAAACCGATGGACCCCTGAAGCTTTGGTTGCGATTCAAG----------------
         Zebrina GF-9   AGAAGTAAACCGATGGACCCCTGAACCTTTGGTTGCGATTCAAG----------------
      Sukali Ndiizi-E   AGAAGTAAACCGATGGACCCCTGAACCTTTGGTTGCGATTCAAG----------------
      Sukali Ndiizi-C   AGAAGTAAACCGATGGACCCCTGAACCTTTGGTTGCGATTCAAG----------------
      Sukali Ndiizi-B   AGAAGTAAACCGATGGACCCCTGAACCCTTGGTTGCGATTCAAG----------------
M. acuminata genomic    AGAAGTAAACCGATGGACCCCTGAAGCTTTGGTTGCGATTCAAGAGGTACTTTTCCTTCA

     M. balbisiana-10   ------------------------------------------------------------
        Gros Michel-2   ------------------------------------------------------------
        Pisang Awak-4   ------------------------------------------------------------
      Sukali Ndiizi-A   ------------------------------------------------------------
         Zebrina GF-8   ------------------------------------------------------------
        Calcutta 4-11   ------------------------------------------------------------
        Gros_Michel-1   ------------------------------------------------------------
      Sukali Ndiizi-H   ------------------------------------------------------------
         Zebrina GF-6   ------------------------------------------------------------
         Zebrina GF-7   ------------------------------------------------------------
        Pisang Awak-5   ------------------------------------------------------------
      Sukali Ndiizi-F   ------------------------------------------------------------
      Sukali Ndiizi-G   ------------------------------------------------------------
         Zebrina GF-9   ------------------------------------------------------------
      Sukali Ndiizi-E   ------------------------------------------------------------
      Sukali Ndiizi-C   ------------------------------------------------------------
      Sukali Ndiizi-B   ------------------------------------------------------------
M. acuminata genomic    TTTTCACTTTGGGACATTGGTTCACTAGTTTCTTGTAAATTTTCATCAAAAATGCTAATT

     M. balbisiana-10   ------------------------------------------------------------
        Gros Michel-2   ------------------------------------------------------------
        Pisang Awak-4   ------------------------------------------------------------
      Sukali Ndiizi-A   ------------------------------------------------------------
         Zebrina GF-8   ------------------------------------------------------------
        Calcutta 4-11   ------------------------------------------------------------
        Gros_Michel-1   ------------------------------------------------------------
      Sukali Ndiizi-H   ------------------------------------------------------------
         Zebrina GF-6   ------------------------------------------------------------
         Zebrina GF-7   ------------------------------------------------------------
        Pisang Awak-5   ------------------------------------------------------------
```

```
      Sukali Ndiizi-F   ------------------------------------------------------------
      Sukali Ndiizi-G   ------------------------------------------------------------
         Zebrina GF-9   ------------------------------------------------------------
      Sukali Ndiizi-E   ------------------------------------------------------------
      Sukali Ndiizi-C   ------------------------------------------------------------
      Sukali Ndiizi-B   ------------------------------------------------------------
 M. acuminata genomic   CAAAATTATATATGAATAATATAATTATCTCAAGTTATTTATATAATTTGGCACTAAGTT

     M. balbisiana-10   ------------------------------------------------------------
        Gros Michel-2   ------------------------------------------------------------
        Pisang Awak-4   ------------------------------------------------------------
      Sukali Ndiizi-A   ------------------------------------------------------------
         Zebrina GF-8   ------------------------------------------------------------
        Calcutta 4-11   ------------------------------------------------------------
        Gros_Michel-1   ------------------------------------------------------------
      Sukali Ndiizi-H   ------------------------------------------------------------
         Zebrina GF-6   ------------------------------------------------------------
         Zebrina GF-7   ------------------------------------------------------------
        Pisang Awak-5   ------------------------------------------------------------
      Sukali Ndiizi-F   ------------------------------------------------------------
      Sukali Ndiizi-G   ------------------------------------------------------------
         Zebrina GF-9   ------------------------------------------------------------
      Sukali Ndiizi-E   ------------------------------------------------------------
      Sukali Ndiizi-C   ------------------------------------------------------------
      Sukali Ndiizi-B   ------------------------------------------------------------
 M. acuminata genomic   AAAAAAAATAAATATCATAATCATATAATGTCCATCCCTACATATTTGGGTCGCTAATTA

     M. balbisiana-10   ------------------------------------------------------------
        Gros Michel-2   ------------------------------------------------------------
        Pisang Awak-4   ------------------------------------------------------------
      Sukali Ndiizi-A   ------------------------------------------------------------
         Zebrina GF-8   ------------------------------------------------------------
        Calcutta 4-11   ------------------------------------------------------------
        Gros_Michel-1   ------------------------------------------------------------
      Sukali Ndiizi-H   ------------------------------------------------------------
         Zebrina GF-6   ------------------------------------------------------------
         Zebrina GF-7   ------------------------------------------------------------
        Pisang Awak-5   ------------------------------------------------------------
      Sukali Ndiizi-F   ------------------------------------------------------------
      Sukali Ndiizi-G   ------------------------------------------------------------
         Zebrina GF-9   ------------------------------------------------------------
      Sukali Ndiizi-E   ------------------------------------------------------------
      Sukali Ndiizi-C   ------------------------------------------------------------
      Sukali Ndiizi-B   ------------------------------------------------------------
 M. acuminata genomic   CATCACAAAGAATAATCAGCATGATGAGATAACATCTTATTGAGGTCCACAGGACATGCA

     M. balbisiana-10   ------------------------------------------------------------
        Gros Michel-2   ------------------------------------------------------------
        Pisang Awak-4   ------------------------------------------------------------
      Sukali Ndiizi-A   ------------------------------------------------------------
         Zebrina GF-8   ------------------------------------------------------------
        Calcutta 4-11   ------------------------------------------------------------
        Gros_Michel-1   ------------------------------------------------------------
      Sukali Ndiizi-H   ------------------------------------------------------------
         Zebrina GF-6   ------------------------------------------------------------
         Zebrina GF-7   ------------------------------------------------------------
        Pisang Awak-5   ------------------------------------------------------------
      Sukali Ndiizi-F   ------------------------------------------------------------
      Sukali Ndiizi-G   ------------------------------------------------------------
         Zebrina GF-9   ------------------------------------------------------------
      Sukali Ndiizi-E   ------------------------------------------------------------
      Sukali Ndiizi-C   ------------------------------------------------------------
      Sukali Ndiizi-B   ------------------------------------------------------------
 M. acuminata genomic   AATAAATCGGCCCCAGATTTGGCTCATGTCTAGGTCTATAGACAAGCAATCAACTTTGTT
```

```
   M. balbisiana-10   ------------------------------------------------------------
      Gros Michel-2   ------------------------------------------------------------
      Pisang Awak-4   ------------------------------------------------------------
    Sukali Ndiizi-A   ------------------------------------------------------------
       Zebrina GF-8   ------------------------------------------------------------
      Calcutta 4-11   ------------------------------------------------------------
      Gros_Michel-1   ------------------------------------------------------------
    Sukali Ndiizi-H   ------------------------------------------------------------
       Zebrina GF-6   ------------------------------------------------------------
       Zebrina GF-7   ------------------------------------------------------------
      Pisang Awak-5   ------------------------------------------------------------
    Sukali Ndiizi-F   ------------------------------------------------------------
    Sukali Ndiizi-G   ------------------------------------------------------------
       Zebrina GF-9   ------------------------------------------------------------
    Sukali Ndiizi-E   ------------------------------------------------------------
    Sukali Ndiizi-C   ------------------------------------------------------------
    Sukali Ndiizi-B   ------------------------------------------------------------
M. acuminata genomic   TCACCTTCAGATGGGTTGCGTGCCTACTCAGGGGAGCAAGGGTGTCTTCATATACACCAA

   M. balbisiana-10   ------------------------------------------------------------
      Gros Michel-2   ------------------------------------------------------------
      Pisang Awak-4   ------------------------------------------------------------
    Sukali Ndiizi-A   ------------------------------------------------------------
       Zebrina GF-8   ------------------------------------------------------------
      Calcutta 4-11   ------------------------------------------------------------
      Gros_Michel-1   ------------------------------------------------------------
    Sukali Ndiizi-H   ------------------------------------------------------------
       Zebrina GF-6   ------------------------------------------------------------
       Zebrina GF-7   ------------------------------------------------------------
      Pisang Awak-5   ------------------------------------------------------------
    Sukali Ndiizi-F   ------------------------------------------------------------
    Sukali Ndiizi-G   ------------------------------------------------------------
       Zebrina GF-9   ------------------------------------------------------------
    Sukali Ndiizi-E   ------------------------------------------------------------
    Sukali Ndiizi-C   ------------------------------------------------------------
    Sukali Ndiizi-B   ------------------------------------------------------------
M. acuminata genomic   AAGATCTTGCAGTTTAAGATTTCTAGATTGATGGTGCTTGAACTGTAGCAAAGCTTTTCA

   M. balbisiana-10   ------------------------------------------------------------
      Gros Michel-2   ------------------------------------------------------------
      Pisang Awak-4   ------------------------------------------------------------
    Sukali Ndiizi-A   ------------------------------------------------------------
       Zebrina GF-8   ------------------------------------------------------------
      Calcutta 4-11   ------------------------------------------------------------
      Gros_Michel-1   ------------------------------------------------------------
    Sukali Ndiizi-H   ------------------------------------------------------------
       Zebrina GF-6   ------------------------------------------------------------
       Zebrina GF-7   ------------------------------------------------------------
      Pisang Awak-5   ------------------------------------------------------------
    Sukali Ndiizi-F   ------------------------------------------------------------
    Sukali Ndiizi-G   ------------------------------------------------------------
       Zebrina GF-9   ------------------------------------------------------------
    Sukali Ndiizi-E   ------------------------------------------------------------
    Sukali Ndiizi-C   ------------------------------------------------------------
    Sukali Ndiizi-B   ------------------------------------------------------------
M. acuminata genomic   TTTTTGGATTAGTGTACTCTTTTCTTTGTGCTTTTTTCAATTGGTTGTCACCAGAGCTTT

   M. balbisiana-10   ------------------------------------------------------------
      Gros Michel-2   ------------------------------------------------------------
      Pisang Awak-4   ------------------------------------------------------------
    Sukali Ndiizi-A   ------------------------------------------------------------
       Zebrina GF-8   ------------------------------------------------------------
      Calcutta 4-11   ------------------------------------------------------------
```

```
        Gros_Michel-1   ------------------------------------------------------------
      Sukali Ndiizi-H   ------------------------------------------------------------
         Zebrina GF-6   ------------------------------------------------------------
         Zebrina GF-7   ------------------------------------------------------------
        Pisang Awak-5   ------------------------------------------------------------
      Sukali Ndiizi-F   ------------------------------------------------------------
      Sukali Ndiizi-G   ------------------------------------------------------------
         Zebrina GF-9   ------------------------------------------------------------
      Sukali Ndiizi-E   ------------------------------------------------------------
      Sukali Ndiizi-C   ------------------------------------------------------------
      Sukali Ndiizi-B   ------------------------------------------------------------
M. acuminata genomic    CCTGAGTTTTGTTGTCTTTAGATATAAATGGTTAGTGATACCAGAATTAGCTGACCACAA

    M. balbisiana-10    ------------------------------------------------------------
        Gros Michel-2   ------------------------------------------------------------
        Pisang Awak-4   ------------------------------------------------------------
      Sukali Ndiizi-A   ------------------------------------------------------------
         Zebrina GF-8   ------------------------------------------------------------
        Calcutta 4-11   ------------------------------------------------------------
        Gros_Michel-1   ------------------------------------------------------------
      Sukali Ndiizi-H   ------------------------------------------------------------
         Zebrina GF-6   ------------------------------------------------------------
         Zebrina GF-7   ------------------------------------------------------------
        Pisang Awak-5   ------------------------------------------------------------
      Sukali Ndiizi-F   ------------------------------------------------------------
      Sukali Ndiizi-G   ------------------------------------------------------------
         Zebrina GF-9   ------------------------------------------------------------
      Sukali Ndiizi-E   ------------------------------------------------------------
      Sukali Ndiizi-C   ------------------------------------------------------------
      Sukali Ndiizi-B   ------------------------------------------------------------
M. acuminata genomic    CTGACTGTAGTACCATATATCACCTTGAAGTGCCTGCTTGAACTTGATTCCTAACAACCC

    M. balbisiana-10    ------------------------------------------------------------
        Gros Michel-2   ------------------------------------------------------------
        Pisang Awak-4   ------------------------------------------------------------
      Sukali Ndiizi-A   ------------------------------------------------------------
         Zebrina GF-8   ------------------------------------------------------------
        Calcutta 4-11   ------------------------------------------------------------
        Gros_Michel-1   ------------------------------------------------------------
      Sukali Ndiizi-H   ------------------------------------------------------------
         Zebrina GF-6   ------------------------------------------------------------
         Zebrina GF-7   ------------------------------------------------------------
        Pisang Awak-5   ------------------------------------------------------------
      Sukali Ndiizi-F   ------------------------------------------------------------
      Sukali Ndiizi-G   ------------------------------------------------------------
         Zebrina GF-9   ------------------------------------------------------------
      Sukali Ndiizi-E   ------------------------------------------------------------
      Sukali Ndiizi-C   ------------------------------------------------------------
      Sukali Ndiizi-B   ------------------------------------------------------------
M. acuminata genomic    AAATATGTCAATGAACCTTTTTGTGTTTTTCCTTAAAAAATTGCATTTCACTTTTCATTT

    M. balbisiana-10    ------------------------------------------------------------
        Gros Michel-2   ------------------------------------------------------------
        Pisang Awak-4   ------------------------------------------------------------
      Sukali Ndiizi-A   ------------------------------------------------------------
         Zebrina GF-8   ------------------------------------------------------------
        Calcutta 4-11   ------------------------------------------------------------
        Gros_Michel-1   ------------------------------------------------------------
      Sukali Ndiizi-H   ------------------------------------------------------------
         Zebrina GF-6   ------------------------------------------------------------
         Zebrina GF-7   ------------------------------------------------------------
        Pisang Awak-5   ------------------------------------------------------------
      Sukali Ndiizi-F   ------------------------------------------------------------
      Sukali Ndiizi-G   ------------------------------------------------------------
```

```
          Zebrina GF-9   ------------------------------------------------------------
       Sukali Ndiizi-E   ------------------------------------------------------------
       Sukali Ndiizi-C   ------------------------------------------------------------
       Sukali Ndiizi-B   ------------------------------------------------------------
  M. acuminata genomic   AAACCTATCTGTTTGATTAGATAATACATTTCAGTATCCAGATCTTTTACATCAGCATCA

      M. balbisiana-10   ------------------------------------------------------------
         Gros Michel-2   ------------------------------------------------------------
         Pisang Awak-4   ------------------------------------------------------------
       Sukali Ndiizi-A   ------------------------------------------------------------
          Zebrina GF-8   ------------------------------------------------------------
         Calcutta 4-11   ------------------------------------------------------------
         Gros_Michel-1   ------------------------------------------------------------
       Sukali Ndiizi-H   ------------------------------------------------------------
          Zebrina GF-6   ------------------------------------------------------------
          Zebrina GF-7   ------------------------------------------------------------
         Pisang Awak-5   ------------------------------------------------------------
       Sukali Ndiizi-F   ------------------------------------------------------------
       Sukali Ndiizi-G   ------------------------------------------------------------
          Zebrina GF-9   ------------------------------------------------------------
       Sukali Ndiizi-E   ------------------------------------------------------------
       Sukali Ndiizi-C   ------------------------------------------------------------
       Sukali Ndiizi-B   ------------------------------------------------------------
  M. acuminata genomic   TGGATTGACAGCATACATTCTGATAGGATTGATGAACTTGGTTGTTGATAAGACTATAAA

      M. balbisiana-10   ------------------------------------------------------------
         Gros Michel-2   ------------------------------------------------------------
         Pisang Awak-4   ------------------------------------------------------------
       Sukali Ndiizi-A   ------------------------------------------------------------
          Zebrina GF-8   ------------------------------------------------------------
         Calcutta 4-11   ------------------------------------------------------------
         Gros_Michel-1   ------------------------------------------------------------
       Sukali Ndiizi-H   ------------------------------------------------------------
          Zebrina GF-6   ------------------------------------------------------------
          Zebrina GF-7   ------------------------------------------------------------
         Pisang Awak-5   ------------------------------------------------------------
       Sukali Ndiizi-F   ------------------------------------------------------------
       Sukali Ndiizi-G   ------------------------------------------------------------
          Zebrina GF-9   ------------------------------------------------------------
       Sukali Ndiizi-E   ------------------------------------------------------------
       Sukali Ndiizi-C   ------------------------------------------------------------
       Sukali Ndiizi-B   ------------------------------------------------------------
  M. acuminata genomic   AAGAAATACTAATTGTTTTCTACATGTGTTCTGGAAAGAGCAATATATTTCTTAGATAAG

      M. balbisiana-10   ------------------------------------------------------------
         Gros Michel-2   ------------------------------------------------------------
         Pisang Awak-4   ------------------------------------------------------------
       Sukali Ndiizi-A   ------------------------------------------------------------
          Zebrina GF-8   ------------------------------------------------------------
         Calcutta 4-11   ------------------------------------------------------------
         Gros_Michel-1   ------------------------------------------------------------
       Sukali Ndiizi-H   ------------------------------------------------------------
          Zebrina GF-6   ------------------------------------------------------------
          Zebrina GF-7   ------------------------------------------------------------
         Pisang Awak-5   ------------------------------------------------------------
       Sukali Ndiizi-F   ------------------------------------------------------------
       Sukali Ndiizi-G   ------------------------------------------------------------
          Zebrina GF-9   ------------------------------------------------------------
       Sukali Ndiizi-E   ------------------------------------------------------------
       Sukali Ndiizi-C   ------------------------------------------------------------
       Sukali Ndiizi-B   ------------------------------------------------------------
  M. acuminata genomic   TTCAATTTAATAGAAGTCACATCACCATTTTTCGTAGCGATAATTATGGTCGTTTTTGAT

      M. balbisiana-10   ------------------------------------------------------------
```

```
      Gros Michel-2   ------------------------------------------------------------
      Pisang Awak-4   ------------------------------------------------------------
    Sukali Ndiizi-A   ------------------------------------------------------------
       Zebrina GF-8   ------------------------------------------------------------
      Calcutta 4-11   ------------------------------------------------------------
      Gros_Michel-1   ------------------------------------------------------------
    Sukali Ndiizi-H   ------------------------------------------------------------
       Zebrina GF-6   ------------------------------------------------------------
       Zebrina GF-7   ------------------------------------------------------------
      Pisang Awak-5   ------------------------------------------------------------
    Sukali Ndiizi-F   ------------------------------------------------------------
    Sukali Ndiizi-G   ------------------------------------------------------------
       Zebrina GF-9   ------------------------------------------------------------
    Sukali Ndiizi-E   ------------------------------------------------------------
    Sukali Ndiizi-C   ------------------------------------------------------------
    Sukali Ndiizi-B   ------------------------------------------------------------
M. acuminata genomic   GATTATGATAAACAGATGATAATTGTATATGGTTCCTGAGCCTTTAATAACAATGTGAAA

   M. balbisiana-10   ------------------------------------------------------------
      Gros Michel-2   ------------------------------------------------------------
      Pisang Awak-4   ------------------------------------------------------------
    Sukali Ndiizi-A   ------------------------------------------------------------
       Zebrina GF-8   ------------------------------------------------------------
      Calcutta 4-11   ------------------------------------------------------------
      Gros_Michel-1   ------------------------------------------------------------
    Sukali Ndiizi-H   ------------------------------------------------------------
       Zebrina GF-6   ------------------------------------------------------------
       Zebrina GF-7   ------------------------------------------------------------
      Pisang Awak-5   ------------------------------------------------------------
    Sukali Ndiizi-F   ------------------------------------------------------------
    Sukali Ndiizi-G   ------------------------------------------------------------
       Zebrina GF-9   ------------------------------------------------------------
    Sukali Ndiizi-E   ------------------------------------------------------------
    Sukali Ndiizi-C   ------------------------------------------------------------
    Sukali Ndiizi-B   ------------------------------------------------------------
M. acuminata genomic   TGGTTTTGACCATGTGACTCTGTTGTTCAATGTTAAGATTTCAAGAGGGGACAAGGGTTA

   M. balbisiana-10   ------------------------------------------------------------
      Gros Michel-2   ------------------------------------------------------------
      Pisang Awak-4   ------------------------------------------------------------
    Sukali Ndiizi-A   ------------------------------------------------------------
       Zebrina GF-8   ------------------------------------------------------------
      Calcutta 4-11   ------------------------------------------------------------
      Gros_Michel-1   ------------------------------------------------------------
    Sukali Ndiizi-H   ------------------------------------------------------------
       Zebrina GF-6   ------------------------------------------------------------
       Zebrina GF-7   ------------------------------------------------------------
      Pisang Awak-5   ------------------------------------------------------------
    Sukali Ndiizi-F   ------------------------------------------------------------
    Sukali Ndiizi-G   ------------------------------------------------------------
       Zebrina GF-9   ------------------------------------------------------------
    Sukali Ndiizi-E   ------------------------------------------------------------
    Sukali Ndiizi-C   ------------------------------------------------------------
    Sukali Ndiizi-B   ------------------------------------------------------------
M. acuminata genomic   TAGGTTGAACCAATGTAAACAAAGTCACTCAAAACCTCATCTTGAATCAAAGTTATCTGA

   M. balbisiana-10   ------------------------------------------------------------
      Gros Michel-2   ------------------------------------------------------------
      Pisang Awak-4   ------------------------------------------------------------
    Sukali Ndiizi-A   ------------------------------------------------------------
       Zebrina GF-8   ------------------------------------------------------------
      Calcutta 4-11   ------------------------------------------------------------
      Gros_Michel-1   ------------------------------------------------------------
    Sukali Ndiizi-H   ------------------------------------------------------------
```

```
        Zebrina GF-6   ------------------------------------------------------------
        Zebrina GF-7   ------------------------------------------------------------
       Pisang Awak-5   ------------------------------------------------------------
     Sukali Ndiizi-F   ------------------------------------------------------------
     Sukali Ndiizi-G   ------------------------------------------------------------
        Zebrina GF-9   ------------------------------------------------------------
     Sukali Ndiizi-E   ------------------------------------------------------------
     Sukali Ndiizi-C   ------------------------------------------------------------
     Sukali Ndiizi-B   ------------------------------------------------------------
M. acuminata genomic   CTCGGAAGATTCGATCATTGTTGACTTCTTATGTATTTTACTTTCATCAGTTTGCTGATT

    M. balbisiana-10   ------------------------------------------------------------
       Gros Michel-2   ------------------------------------------------------------
       Pisang Awak-4   ------------------------------------------------------------
     Sukali Ndiizi-A   ------------------------------------------------------------
        Zebrina GF-8   ------------------------------------------------------------
       Calcutta 4-11   ------------------------------------------------------------
       Gros_Michel-1   ------------------------------------------------------------
     Sukali_Ndiizi-H   ------------------------------------------------------------
        Zebrina GF-6   ------------------------------------------------------------
        Zebrina GF-7   ------------------------------------------------------------
       Pisang Awak-5   ------------------------------------------------------------
     Sukali Ndiizi-F   ------------------------------------------------------------
     Sukali Ndiizi-G   ------------------------------------------------------------
        Zebrina GF-9   ------------------------------------------------------------
     Sukali Ndiizi-E   ------------------------------------------------------------
     Sukali Ndiizi-C   ------------------------------------------------------------
     Sukali Ndiizi-B   ------------------------------------------------------------
M. acuminata genomic   ATGCTTATGCTTACTTGCTTAAATCTAAATTTTTTGAACAAGAACTTCTATATAAGAAAT

    M. balbisiana-10   ------------------------------------------------------------
       Gros Michel-2   ------------------------------------------------------------
       Pisang Awak-4   ------------------------------------------------------------
     Sukali Ndiizi-A   ------------------------------------------------------------
        Zebrina GF-8   ------------------------------------------------------------
       Calcutta 4-11   ------------------------------------------------------------
       Gros_Michel-1   ------------------------------------------------------------
     Sukali_Ndiizi-H   ------------------------------------------------------------
        Zebrina GF-6   ------------------------------------------------------------
        Zebrina GF-7   ------------------------------------------------------------
       Pisang Awak-5   ------------------------------------------------------------
     Sukali Ndiizi-F   ------------------------------------------------------------
     Sukali Ndiizi-G   ------------------------------------------------------------
        Zebrina GF-9   ------------------------------------------------------------
     Sukali Ndiizi-E   ------------------------------------------------------------
     Sukali Ndiizi-C   ------------------------------------------------------------
     Sukali Ndiizi-B   ------------------------------------------------------------
M. acuminata genomic   GCCATATTCAAGTCACAATTATGGTTCAAAGACTCAATCAAAGAAAGGAGTAGGAGTCTG

    M. balbisiana-10   ------------------------------------------------------------
       Gros Michel-2   ------------------------------------------------------------
       Pisang Awak-4   ------------------------------------------------------------
     Sukali Ndiizi-A   ------------------------------------------------------------
        Zebrina GF-8   ------------------------------------------------------------
       Calcutta 4-11   ------------------------------------------------------------
       Gros_Michel-1   ------------------------------------------------------------
     Sukali_Ndiizi-H   ------------------------------------------------------------
        Zebrina GF-6   ------------------------------------------------------------
        Zebrina GF-7   ------------------------------------------------------------
       Pisang Awak-5   ------------------------------------------------------------
     Sukali Ndiizi-F   ------------------------------------------------------------
     Sukali Ndiizi-G   ------------------------------------------------------------
        Zebrina GF-9   ------------------------------------------------------------
     Sukali Ndiizi-E   ------------------------------------------------------------
```

```
     Sukali Ndiizi-C   ------------------------------------------------------------
     Sukali Ndiizi-B   ------------------------------------------------------------
M. acuminata genomic   TTGCCCCAACTTGAAATTAAGAACATCACTTCAATTAATATATTAAAACTATGATTGGCT

    M. balbisiana-10   ------------------------------------------------------------
       Gros Michel-2   ------------------------------------------------------------
       Pisang Awak-4   ------------------------------------------------------------
     Sukali Ndiizi-A   ------------------------------------------------------------
        Zebrina GF-8   ------------------------------------------------------------
       Calcutta 4-11   ------------------------------------------------------------
       Gros_Michel-1   ------------------------------------------------------------
     Sukali Ndiizi-H   ------------------------------------------------------------
        Zebrina GF-6   ------------------------------------------------------------
        Zebrina GF-7   ------------------------------------------------------------
       Pisang Awak-5   ------------------------------------------------------------
     Sukali Ndiizi-F   ------------------------------------------------------------
     Sukali Ndiizi-G   ------------------------------------------------------------
        Zebrina GF-9   ------------------------------------------------------------
     Sukali Ndiizi-E   ------------------------------------------------------------
     Sukali Ndiizi-C   ------------------------------------------------------------
     Sukali Ndiizi-B   ------------------------------------------------------------
M. acuminata genomic   AGTAAGTCAACCTAATACTAGTTGACATTTTCTTAAAAAATAATTGATTTTGGTGTTCTT

    M. balbisiana-10   ---------------------------------------------------------...
       Gros Michel-2   ---------------------------------------------------------...
       Pisang Awak-4   ---------------------------------------------------------...
     Sukali Ndiizi-A   ---------------------------------------------------------...
        Zebrina GF-8   ---------------------------------------------------------...
       Calcutta 4-11   ---------------------------------------------------------...
       Gros_Michel-1   ---------------------------------------------------------...
     Sukali Ndiizi-H   ---------------------------------------------------------...
        Zebrina GF-6   ---------------------------------------------------------...
        Zebrina GF-7   ---------------------------------------------------------...
       Pisang Awak-5   ---------------------------------------------------------...
     Sukali Ndiizi-F   ---------------------------------------------------------...
     Sukali Ndiizi-G   ---------------------------------------------------------...
        Zebrina GF-9   ---------------------------------------------------------...
     Sukali Ndiizi-E   ---------------------------------------------------------...
     Sukali Ndiizi-C   ---------------------------------------------------------...
     Sukali Ndiizi-B   ---------------------------------------------------------...
M. acuminata genomic   AACTTGGTTGAGTACAATAACTTGTGAGAAATTCTAAGAAACTCATTAAAACATACT...
```

Alignment of CENH3 genomic sequence to CENH3 gene transcripts
